# Maternal and neonatal safety outcomes after SAR-CoV-2 vaccination during pregnancy: a systematic review and meta-analysis

**DOI:** 10.1186/s12884-022-04884-9

**Published:** 2022-07-21

**Authors:** Abdulrahman Ibrahim Hagrass, Hossam Waleed Almadhoon, Mohammed Al-kafarna, Bashar Khaled Almaghary, Anas Zakarya Nourelden, Ahmed Hashem Fathallah, Mohammed Tarek Hasan, Yasmine Adel Mohammed, Aya Osama Al-Nabahin, Dalia Sami Wafi, Islam Osama Ismail, Yaser Aref Hamam, Reem Sayad, Mohamed Hamouda, Mohamed Sayed Zaazouee, Khaled Mohamed Ragab

**Affiliations:** 1 Faculty of Medicine, Al-Azhar University, Cairo, Egypt; 2 Faculty of Dentistry, Al-Azhar University, Gaza, Palestine; 3 Faculty of Pharmacy, Al-Azhar University, Gaza, Palestine; 4 Faculty of Medicine, Minia University, Minia, Egypt; 5 Faculty of Medicine, Assiut University, Assiut, Egypt; 6 Department of Clinical Nutrition, Faculty of Applied Medical Sciences, Al-Azhar University, Gaza, Palestine; 7 Faculty of Medicine, Al-Quds University, Abu-Dies, Jerusalem, Israel; 8 Faculty of Medicine, Al-Azhar University, Assiut, Egypt

**Keywords:** COVID-19 vaccine, Pregnancy, Maternal, Neonatal, Safety

## Abstract

**Background and objective:**

More than five million individuals died because of problems connected to COVID-19. SARS-Cov-2 poses a particular challenge to expectant mothers, who comprise one of the most vulnerable segments of the population. Our aim is to demonstrate the maternal and neonatal safety of the COVID-19 vaccine during pregnancy.

**Methods:**

We searched PubMed, Cochrane Library, Scopus, Web of Science (WOS), Embase, Ovid, MedRxiv, and BioRxiv databases from inception till December 2021 and then updated it in April 2022. Additionally, we searched ClinicalTrials.gov, Research Square and grey literature. Cohort, case–control studies, and randomized controlled trials detecting the safety of the Covid-19 vaccine during pregnancy were included. We used the Cochrane tool and Newcastle–Ottawa Scale to assess the risk of bias of the included studies and the GRADE scale to assess the quality of evidence. A meta-analysis was conducted using review manager 5.4.

**Results:**

We included 13 studies with a total number of 56,428 patients. Our analysis showed no statistically significant difference in the following outcomes: miscarriage (1.56% vs 0.3%. RR 1.23; 95%CI 0.54 to 2.78); length of maternal hospitalization (MD 0.00; 95%CI -0.08 to 0.08); puerperal fever (1.71% vs 1.1%. RR 1.04; 95%CI 0.67 to 1.61); postpartum hemorrhage (4.27% vs 3.52%. RR 0.84; 95%CI 0.65 to 1.09); instrumental or vacuum-assisted delivery (4.16% vs 4.54%. RR 0.94; 95%CI 0.57 to 1.56); incidence of Apgar score ≤ 7 at 5 min (1.47% vs 1.48%. RR 0.86; 95%CI 0.54 to 1.37); and birthweight (MD -7.14; 95%CI -34.26 to 19.99).

**Conclusion:**

In pregnancy, the current meta-analysis shows no effect of SAR-CoV-2 vaccination on the risk of miscarriage, length of stay in the hospital, puerperal fever, postpartum hemorrhage, birth weight, or the incidence of an Apgar score of ≤ 7 at 5 min.

**Supplementary Information:**

The online version contains supplementary material available at 10.1186/s12884-022-04884-9.

## Introduction

SARS-CoV-2 pandemic had negative consequences and presented unprecedented obstacles that harmed people's physical and mental health around the world [1]. As of June 1, 2022, it resulted in over 527 million illnesses and over 6 million deaths worldwide [2]. In the absence of a cure, COVID-19 vaccination has proven to be an effective way to stop the pandemic from spreading [3]. Almost every country had implemented a COVID-19 vaccination programme by July 2021 [2]. According to preliminary findings, the present vaccinations are protective against the current variants [4, 5]. Pregnant women are among the most vulnerable groups to SARS-Cov-2 [6–12]. Therefore, many health authorities considered pregnancy as a risk factor for COVID-19 severity [13]. And other organizations are concerned with mother and fetus health [14]. There is a suggestion that pregnant women infected with COVID-19 are more prone to pregnancy consequences. COVID-19 infected pregnant are more susceptible to experiencing pregnancy-induced cardiovascular problems like hypertension and thrombosis and other problems like premature birth [15]. So, there is an urgency for evidence about COVID-19 immunization during pregnancy due to the vulnerability of this population. COVID-19 severity in pregnancy may be attributed to pregnancy immunity changes and lung volume decrease [16–18].

The scientific community had doubts about the transplacental antibody quantity transfer following the SARS-Cov-2 vaccine [19]. Following 14 days of immunization, an antibody against COVID-19 was isolated from umbilical blood samples. After Pfizer–BioNTech COVID-19 vaccine single dosage [20]. Another study suggests maternal immunization should be earlier than three weeks before delivery to allow SARS-Cov-2 antibody transfer to the fetus. Earlier immunization, especially in the third trimester, may positively correlate with infant immunity [21]. But the accurate time of vaccination during pregnancy is still controversial.

Pregnant women are regularly excluded from new drug and vaccine trials because of fears about the fetus. Phase iii safety and efficacy trials on SARS-CoV-2 vaccines did not include pregnant females in their population, so our knowledge regarding vaccination during pregnancy is still limited [22]. This knowledge gap poses a challenge for obstetricians and gynecologists in counseling pregnant women about the vaccine [22]. Pregnant acceptability of the vaccine is lower than in the case of non-pregnant. And public trust in vaccination safety and efficacy is the main factor in vaccine uptake [23]. Good evidence can help to increase vaccine acceptance. As SARS-CoV-2 is vulnerable, many health ministries provide vaccines to pregnant women despite a lack of evidence for potential reliable effects. We aim to assess the safety profile of COVID-19 vaccine uptake in pregnancy.

## Methods

Our systematic review and meta-analysis was conducted according to the Cochrane handbook [24], and the PRISMA guideline [25] and registered with PROSPERO (CRD42022334425).

### Literature search and data collection

We searched PubMed, Cochrane Library, Scopus, web of science (WOS), Embase, Ovid, MedRxiv, and BioRxiv databases. We also searched the results of published protocols (ClinicalTrials.gov) and preprinted papers (Research Square). We complemented the databases search with a manual search of grey literature (www.opengrey.eu/). No filters were used, and all identified results were checked against the eligibility criteria. We searched the literature from inception till December 2021 and then updated it in April 2022. The details of the used search strategy are summarized in supplementary file 1.

### Eligibility criteria

Two independent researchers (H. W. Madhoon, M. T. Hasan) reviewed the references using previously established eligibility criteria. We used EndNote software to collect the results of the databases search. We removed the duplicates using the built-in duplicate removal feature before exporting the de-duplicated studies to Microsoft Excel (2021 Edition: Microsoft Corp, Redmond, WA) to screen the title and abstract, and then the full text. Our eligibility criteria were 1) population: pregnant women; 2) intervention: COVID-19 vaccine. 3) comparators: unvaccinated women; 4) outcome: safety outcomes. 5) study design eligible: cohort, case–control, and randomized controlled trials (RCTs).

### Methodological quality assessment

We assessed the included RCTs for methodological bias risk according to the Cochrane tool. [24] The tool consists of domains including randomization process, allocation of study arms, blinding of participants and investigators, outcome assessment blinding, outcomes, reporting bias, and other biases. Judgment is based upon the risk of bias which can be low, high, or unclear. Newcastle–Ottawa Scale (NOS) [26] was used to assess non-RCTs studies. It includes three main domains 1) selection (cases and control definition, cases and controls selection) maximum of four stars, 2) comparability (are cases and controls comparable or not) maximum of two stars, 3) exposure (for what degree we are confident that our population is exposed to the exposure) maximum three stars. This work was done separately by four authors (Y. A. Mohammed, A. O. Al-Nabahin, D. S. Wafi, and R. Sayad). A fifth author (A.I. Hagrass) was consulted to resolve any conflicts. The GRADE methodology (GRADEpro, version 20. McMaster University, 2013) was used to assess the quality of evidence of the analyzed outcomes [27].

### Data extraction

In an excel sheet, we retrieved the following information: 1) Summary: study ID, title, study design, country, and implementation date, participants and key inclusion/exclusion requirements, study arms, follow-up length, and conclusion. 2) Characteristics of the sampled population at the start; age, gender, pre-gravid BMI (kg/m2 maternal comorbidities, first vaccine dose GA, vaccine type, the vaccination-birth interval in days, trimester at vaccination, self-reported ethnicity, obesity (BMI ≥ 30 kg/m2), antenatal medication, prior SARS-CoV-2 infection, gestational age (Weeks), days elapsed between the second vaccination dosage and the collection of samples and from symptom onset to sample collection, pyrexia during the next 48 h of vaccination, CDC Risk Factor Count, flu Vaccinations in the Last 5 Years and other data. 3) Study outcomes as described below. Four independent authors (M. Al-kafarna, B. K. Almaghary, A. H. Fathallah, M. T. Hasan) extracted data; a fifth author (A.I. Hagrass) was consulted to resolve any conflicts.

### Study Outcomes

The maternal outcomes include the length of maternal hospitalization, puerperal fever, postpartum hemorrhage, placental abruption, suspected chorioamnionitis, and maternal intensive care unit (ICU) admission. The Obstetric outcomes include Miscarriage, Birth type, Gestational age at delivery, and Preterm birth. The neonatal outcomes include Neonatal unit admission, Apgar ≤ 7 at 5 min, Birth Weight, and Composite adverse neonatal outcomes. Composite adverse neonatal outcomes are a composite of any of the following events: intrauterine fetal death, 5-min Apgar score < 7, NICU admission, and neonatal asphyxia.

### Data synthesis

We analyzed the extracted data using Review Manager (RevMan) software version 5.4. We used the risk ratio (RR) and 95% confidence interval (CI) in the case of dichotomous data. We pooled a 95% confidence interval (CI) and mean difference (MD) if the data were continuous. We reported significance if the p-value was less than 5%. When the Chi-Square P value was less than 0.1 and the I^2^-value was greater than 50%, the data were deemed heterogeneous. We selected the random-effect model if the data were heterogeneous and the fixed-effect model if it wasn't. Subgroup analysis was performed based on the study design.

## Results

### Literature search

**Fig. 1:**
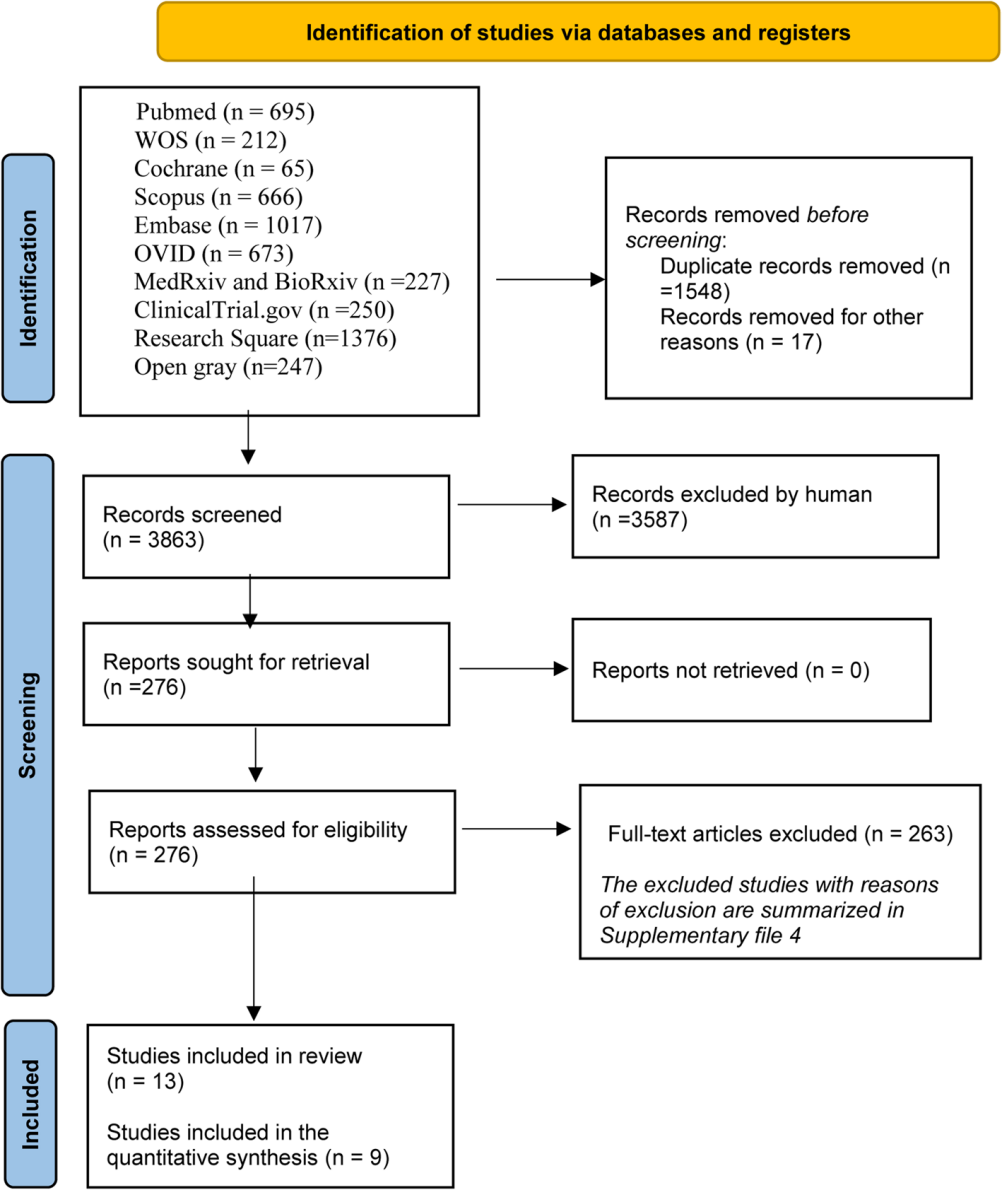
PRISMA flow chart

The literature search strategy retrieved 2386 citations after the removal of duplications. After we did the title and abstract screening, 276 articles were reliable for full-text screening. 13 studies [20, 28–39] were included in qualitative synthesis for matching our inclusion criteria, and nine studies [20, 28–30, 34, 36–39] were included in the quantitative synthesis (Fig. 1; Supplementary File 2). After checking the sources of included research, no missing publications were discovered.

### Characteristics of included studies

We included 13 studies [20, 28–39] in our study in a total number of 56,428 patients; three [28, 29, 34] of them are RCTs, one [31] is case–control, and nine [20, 30, 32, 33, 36–39] are cohorts. During the course of the included studies (in late 2020 and early 2021), the most common variants were Epsilon (B.1.427—B.1.429) and Alpha (B.1.1.7) variants [40]. Some studies gave BNT162b2 mRNA COVID-19 vaccine, and others gave Moderna vaccine or ChAdOx1 nCoV-19 Vaccine, so we included any study using a vaccine to COVID-19 in pregnant women as intervention and unvaccinated pregnant women as a control in our inclusion criteria. Side effects data was detected by direct observation from the investigator in RCTs. While in retrospective cohort studies, it was detected by hospital records review, then asking the women in postnatal unit about their immunization status with comparing their answers to the hospital records. Tables 1 and 2

**Table 1:** Summary of the included studies

Study ID	Study design, country and time of realization	Participants	Intervention group	Control group	Main inclusion criteria	Exclusion criteria	Primary outcomes
Beharier 2021 [20]	Cohort, Jerusalem, between April 2020 and March 2021	1094	Vaccinated group during pregnancy	Unvaccinated non infected controls	Age of 18 years or older and a willingness to participate and provide informed consent	Pregnant women with active maternal COVID-19 disease at delivery	
Blakeway 2021 [30]	Cohort, London, United Kingdom, between March 1, 2020, and July 4, 2021	1328	At least 1 dose during pregnancy	Did not receive a vaccine during pregnancy	Pregnant women with known vaccination status and complete maternal and fetal outcome data	women who were vaccinated entirely (i.e., all doses) before pregnancy or after birth or women who had pregnancies complicated by fetal aneuploidy or genetic syndromes	COVID-19 vaccine uptake during pregnancy among women eligible for vaccination
Butt 2021 [31]	Case–control, Qatar, Between December 20, 2020, and May 30, 2021	2020	PCR positive of Pregnant women	PCR negative of Pregnant women	All women presented to Hamad Medical Corporation between December 20, 2020, and May 30, 2021, with confirmed pregnancies	who were tested for SARS-CoV-2 by RT-PCR prior to pregnancy and those who had no SARS-CoV-2 testing done between December 20, 2020, and May 30, 2021	overall vaccine effectiveness > 14 days after the second dose of the vaccine, we also determined vaccine effectiveness > 14 days after the first dose up to the date of the second dose
Collier 2021 [32]	Cohort, Jerusalem, from December 2020 through March 2021	103	Pregnant Vaccinated women	Pregnant Unvaccinated and infected women	Pregnant, lactating, and non-pregnant women aged 18 to 45 years who were vaccinated or infected	_	SARS-CoV-2 receptor binding domain binding, neutralizing, and functional non-neutralizing antibody responses from pregnant, lactating, and nonpregnant women were assessed following vaccination
Dagan 2021 [33]	Cohort Jerusalem, between 20 December 2020 and 3 June 2021	21,722	Pregnant Vaccinated women	Pregnant Unvaccinated and infected women	Pregnancy, age of 16 years or older, continuous membership in CHS for 1 complete year, no previous positive SARS-CoV-2 PCR test, no previous SARS-CoV-2 vaccination, not residing in long-term care facilities	Individuals with missing data (only relevant for the body mass index and living area variables)	documented SARS-CoV-2 infection, symptomatic SARS-CoV-2 infection (COVID-19), COVID-19-related hospitalization; severe COVID-19, and COVID-19-related death
Kharbanda 2021 [35]	Cohort, USA, from December 15, 2020, through June 28, 2021	21,267	Ongoing pregnancy periods (vaccinated women)	Spontaneous abortions (vaccinated women)	_	_	_
Rottenstreich 2021 [36]	Cohort, Jerusalem, between January and April 2021	1775	Covid‐19 vaccinated Pregnant women	Covid‐19 Unvaccinated Pregnant women	All women aged 18 years or older, with no documented previous positive PCR test, who delivered between 19 January 2021 (when the first vaccinated women gave birth) and 27 April 2021	women with current or previous Covid‐19 disease	chorioamnionitis, postpartum hemorrhage, endometritis, blood transfusion, a cesarean delivery (CD), ICU admission, and a maternal hospital length of stay of > 5 days for vaginal delivery and > 7 days for CD
Shanes 2021 [37]	Cohort, USA	200	Pregnant Vaccinated women	Pregnant Unvaccinated women	_	_	_
Theiler 2021 [38]	Cohort, USA	2002	Covid‐19 vaccinated Pregnant women	Covid‐19 Unvaccinated Pregnant women	All patients aged 16 to 55 years with a delivery event between December 10, 2020, and April 19, 2021, at a hospital within the Mayo Clinic Health System	Patients who opted out to use their medical records for research if their delivery occurred in Minnesota	(1) maternal death during hospitalization; (2) intrapartum neonatal death within 7 days of birth; (3) hypoxic-ischemic encephalopathy; (4) uterine rupture; (5) unplanned maternal ICU admission; (6) return to the operating room within 72 h of delivery; (7) postpartum hemorrhage with blood transfusion; (8) third- or fourth-degree laceration; (9) 5-min Apgar score of < 7; (10) admission to the neonatal ICU within 1 day of birth for > 1 day; or (11) neonatal birth trauma
Wainstock 2021 [39]	Cohort, Jerusalem, between January and June 2021	4,860	Covid‐19 vaccinated Pregnant women	Covid‐19 Unvaccinated Pregnant women	_	_	_
Pfizer BioNTech C4591001	RCT	37,706	BNT162b2 (30 μg)	Placebo	_	_	vaccine efficacy
Moderna mRNA-1273-P301	RCT	30,418	Moderna COVID-19 Vaccine mRNA-1273	Placebo	_	_	_
COV003 (Brazil)	RCT, Brazil	6753	ChAdOx1 nCoV-19	MenACWY "control vaccine" (first dose), Saline (second dose)	Adults aged 18 years and older	_	_

**Table 2:** Baseline characteristics for included studies

ID	study groups	sample size	Maternal Age, y, (Mean ± SD)	Pregravid BMI (kg/m2), (Mean ± SD)	Maternal comorbidities, N (%)				Gender, N (%)		Trimester at vaccination, n (%)	
					Hypertensive disorders	Diabetes or gestational diabetes	Asthma	Smoker	Male	Female	First	Second
Beharier 2021 [20]	Vaccinated group during pregnancy	92	31.7 ± 5.8	24.2 ± 5.2	1 (1.1)	8 (8.7)	2 (2.2)	6 (6.5)	45 (49.5)	46 (50.5)		
	Unvaccinated non infected controls	66	31.6 ± 5.8	25.7 ± 6.5	1 (1.5)	9 (13.6)	1 (1.5)	4 (6.6)	31 (47.7)	34 (52.3)		
Blakeway 2021 [30]	At least 1 dose during pregnancy	140	34.33 ± 4.94	24.2 ± 4.4	13 (9.2)			1 (0.7)			0 (0.0)	20 (14.2)
	Did not receive a vaccine during pregnancy	1188	33 ± 4.45	24.8 ± 4.8	46 (3.9)			27 (2.3)				
Butt 2021 [31]	PCR positive	393	30.67 ± 5.21									
	PCR negative	862	31.33 ± 5.2									
Collier 2021 [32]	Pregnant Vaccinated women	30	34.33 ± 3.11								5 (17%)	15 (50%)
	Pregnant Unvaccinated and infected women	22	31.67 ± 6.34									
Dagan 2021 [33]	Pregnant Vaccinated women	10,861	29.67 ± 5.19		40 (0.4%)	52 (0.5%)	372 (3.4%)	643 (5.9%)			2,814 (26%)	5,242 (48%)
	Pregnant Unvaccinated and infected women	10,861	29.67 ± 5.19		34 (0.3%)	57 (0.5%)	388 (3.6%)	701 (6.5%)				
Kharbanda 2021 [35]	Ongoing pregnancy periods (vaccinated women)	20,139										
	Spontaneous abortions (vaccinated women)	1128										
Rottenstreich 2021 [36]	Covid‐19 vaccinated Pregnant women	712	30.6 ± 5.8		10 (1.4%)	45 (6.3%)						
	Covid‐19 Unvaccinated Pregnant women	1063	29.5 ± 6		19 (1.8%)	45 (4.2%)						
Shanes 2021 [37]	Pregnant Vaccinated women	84	33.7 ± 3.1									
	Pregnant Unvaccinated women	116	32.5 ± 4.8									
Theiler 2021 [38]	Covid‐19 vaccinated Pregnant women	140	31.8 ± 3.7		6 (4.3)		15 (10.7)	0				
	Covid‐19 Unvaccinated Pregnant women	1862	30.5 ± 5.2		64 (3.4)		206 (11.1)	196 (10.5)				
Wainstock 2021 [39]	Covid‐19 vaccinated Pregnant women	913	30.6 ± 5.3		50 (5.5)	63 (6.9)						
	Covid‐19 Unvaccinated Pregnant women	3486	28.2 ± 5.7		165 (4.7)	187 (5.4)						
Pfizer BioNTech C4591001	BNT162b2 (30 μg)	18,860							9639 (51.1)	9221 (48.9)		
	Placebo	18,846							9436 (50.1)	9410 (49.9)		
Moderna mRNA-1273-P301	Moderna COVID-19 Vaccine mRNA-1273	15,208										
	Placebo	15,210										
COV003 (Brazil)	ChAdOx1nCoV-19	3414		26.07 ± 4.45		141 (4.1%)			1478 (43.3)	1936 (56.7%)		
	MenACWY "control vaccine" (first dose), Saline (second dose)	3339		26.17 ± 4.6		113 (3.4%)			1500 (44.9)	1839 (55.1%)		

ID	study groups	sample size	Maternal Age, y, (Mean ± SD)	Pregravid BMI (kg/m2), (Mean ± SD)	Trimester at vaccination, n (%)	Self-reported ethnicity, n (%)								Obesity (BMI ≥ 30 kg/m2), n (%)
					Third	Caucasian	Afro-Caribbean	Asian	Mixed	Not reported	White	Other	Hispanic or Latina	
Beharier 2021 [20]	Vaccinated group during pregnancy	92	31.7 ± 5.8	24.2 ± 5.2										
	Unvaccinated non infected controls	66	31.6 ± 5.8	25.7 ± 6.5										
Blakeway 2021 [30]	At least 1 dose during pregnancy	140	34.33 ± 4.94	24.2 ± 4.4	121 (85.8)	80 (56.7)	18 (12.8)	5 (3.5)	13 (9.2)	25 (17.7)				15 (11.5)
	Did not receive a vaccine during pregnancy	1188	33 ± 4.45	24.8 ± 4.8		551 (46.4)	204 (17.2)	101 (8.5)	156 (13.1)	175 (14.7)				173 (17.4)
Butt 2021 [31]	PCR positive	393	30.67 ± 5.21											
	PCR negative	862	31.33 ± 5.2											
Collier 2021 [32]	Pregnant Vaccinated women	30	34.33 ± 3.11		10 (33%)		0	3 (11)	1 (4)		24 (86)		1 (4)	
	Pregnant Unvaccinated and infected women	22	31.67 ± 6.34				5 (28)	0	3 (17)		10 (56)		4 (21)	
Dagan 2021 [33]	Pregnant Vaccinated women	10,861	29.67 ± 5.19		2,805 (26%)									1,048 (9.6%)
	Pregnant Unvaccinated and infected women	10,861	29.67 ± 5.19											1,019 (9.4%)
Kharbanda 2021 [35]	Ongoing pregnancy periods (vaccinated women)	20,139					715 (3.8)	4433 (12.3)			7571 (9.3)	2213 (7.8)	5207 (6.0)	
	Spontaneous abortions (vaccinated women)	1128					48 (4.4)	262 (12.9)			373 (8.7)	123 (8.6)	322 (7.4)	
Rottenstreich 2021 [36]	Covid‐19 vaccinated Pregnant women	712	30.6 ± 5.8											101 (14.2%)
	Covid‐19 Unvaccinated Pregnant women	1063	29.5 ± 6											140 (13.2%)
Shanes 2021 [37]	Pregnant Vaccinated women	84	33.7 ± 3.1											
	Pregnant Unvaccinated women	116	32.5 ± 4.8											
Theiler 2021 [38]	Covid‐19 vaccinated Pregnant women	140	31.8 ± 3.7				3 (2.2)	6 (4.3)			128 (92.1)	1	5 (3.6)	
	Covid‐19 Unvaccinated Pregnant women	1862	30.5 ± 5.2				99 (5.4)	89 (4.8)			1528 (82.9)	18	173 (9.5)	
Wainstock 2021 [39]	Covid‐19 vaccinated Pregnant women	913	30.6 ± 5.3											152 (16.6)
	Covid‐19 Unvaccinated Pregnant women	3486	28.2 ± 5.7											549 (15.7)
Pfizer BioNTech C4591001	BNT162b2 (30 μg)	18,860					1729 (9.2)	801 (4.2)	449 (2.4)	93 (0.5)	15,636 (82.9)		5266 (27.9)	6556 (34.8)
	Placebo	18,846					1763 (9.4)	807 (4.3)	406 (2.2)	115 (0.6)	15,630 (82.9)		5277 (28.0)	6662 (35.3)
Moderna mRNA-1273-P301	Moderna COVID-19 Vaccine mRNA-1273	15,208					1562 (10.3)	653 (4.3)	315 (2.1)		12,032 (79.2)	321 (2.1)	3121 (20.6)	
	Placebo	15,210					1528 (10.1)	732 (4.8)	319 (2.1)		11,990 (79.1)	315 (2.1)	3112 (20.5)	
COV003 (Brazil)	ChAdOx1nCoV-19	3414		26.07 ± 4.45			337 (9.9%)	83 (2.4%)	704 (20.6%)		2273 (66.6%)	17 (0.5%)		
	MenACWY "control vaccine" (first dose), Saline (second dose)	3339		26.17 ± 4.6			336 (10.1%)	66 (2.0%)	670 (20.1%)		2249 (67.4%)	18 (0.5%)		

### Quality assessment

The included cohort studies [20, 30, 32, 33, 35–39] had a score range of 8 to 9 stars out of 9, with the majority of studies scoring 8 (Supplementary Table 3A). Therefore, all studies can be classified as having high quality. Butt et al. [31] is a case–control study of good quality (Supplementary Table 3B). Three studies [28, 29, 34] are RCTs and can be classified as low to unclear risk of bias (Supplementary Table 3C). All three RCTs have sponsors, and we considered it a conflict of interest and a high risk of bias. There was insufficient information about the sequence generation, allocation concealment process, or detection bias in Moderna [34] and COV003 (Brazil) [29]. The GRADE tool revealed low to very low overall evidence quality (Supplementary file 4).

### Qualitative synthesis

Butt et al. [31] showed that the mRNA vaccines are effective after the second dose by 67.7% against SARS-CoV-2 infection in pregnant women, therefore they recommended that pregnant women can be included in vaccination campaigns because of the great level of protection provided by mRNA vaccines. Meanwhile, Kharbanda et al. [35] established according to their sample size that 8.0 percent of ongoing pregnancy periods received a COVID-19 immunization within 28 days of the index date, compared to 8.6 percent of spontaneous abortions. When compared to ongoing pregnancies, spontaneous abortions had no higher odds of receiving a vaccination in the previous 28 days (adjusted odds ratio, 1.02; 95%CI, 0.96 to 1.08). The findings for mRNA-1273 and BNT162b2 were consistent among gestational age groups. In addition, Dagan et al. [33] found that the BNT162b2 mRNA COVID-19 vaccination is extremely successful in pregnant women against the circulating variations at the time of the study, with vaccine efficacy equivalent to that estimated in the general population. Moreover, Coiller et al. [32] established that pregnant women were immunogenic after receiving a COVID-19 mRNA vaccine, and vaccine-elicited antibodies were transferred to newborn cord blood and breast milk. Vaccination of pregnant or non-pregnant women induces anti-SARS-CoV-2 cross-reactive antibody and T-cell responses.

### Quantitative synthesis

#### Maternal outcomes

##### Length of maternal hospitalization (days)

**Fig. 2:**
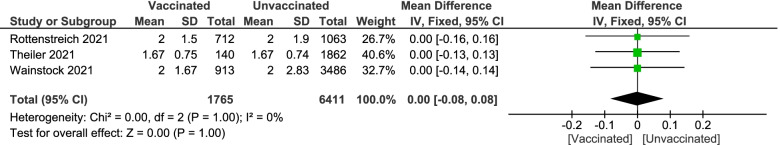
Forest plot of length of maternal hospitalization (days)

Pooled studies [36, 38, 39] measured length of maternal hospitalization revealed no significant difference between vaccinated women and unvaccinated women (MD 0.00; 95%CI -0.08 to 0.08; *P* = 1), pooled results were homogenous (*P* = 1; I.^2^ = 0%) Fig. 2.

##### Intrapartum & postpartum complications

**Fig. 3:**
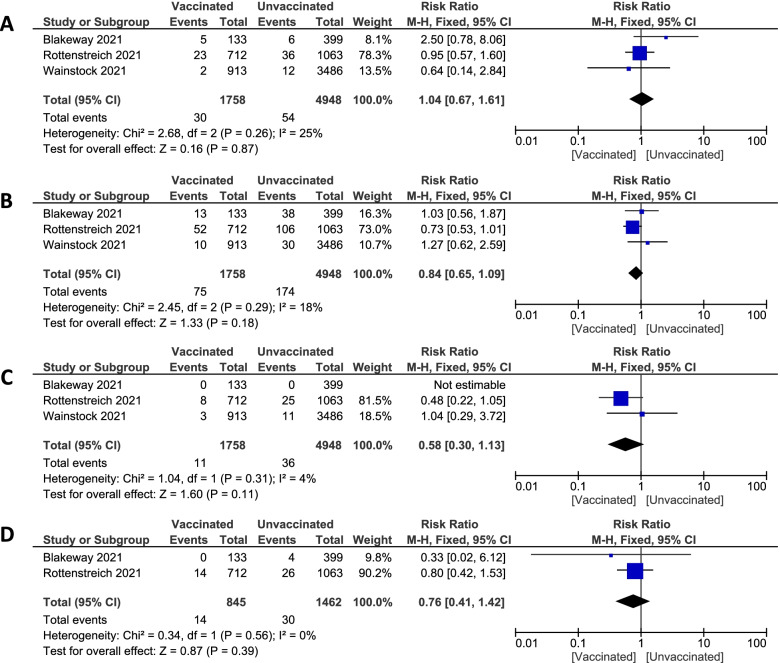
Forest plot of intrapartum and postpartum complications; (**A**) puerperal fever, (**B**) postpartum hemorrhage, (**C**) placental abruption, (**D**) Suspected chorioamnionitis

1. **Puerperal fever:**
  Pooled studies [30, 36, 39] regarding puerperal fever established no statistically significant difference in the total number of pregnant women having puerperal fever between vaccinated pregnant women and unvaccinated pregnant women (1.71% vs. 1.1%. RR 1.04; 95% CI 0.67 to 1.61; P = 0.87), pooled results were homogenous (P = 0.26; I^2^ = 25%) Figure 3A.
2. **Postpartum hemorrhage**
  Pooled studies [30, 36, 39] recorded postpartum hemorrhage showed no significant difference between vaccinated and unvaccinated pregnant women (4.27% vs. 3.52%. RR 0.84; 95% CI 0.65 to 1.09; P = 0.18), pooled results were homogenous (P = 0.29; I^2^ = 18%). Figure 3B
3. **Placental abruption**
  Pooled studies [30, 36, 39] documented placental abruption revealed no statistically significant difference in the prevalence of placental abruption between vaccinated and unvaccinated pregnant women (0.63% vs. 0.73%. RR 0.58; 95% CI 0.30 to 1.13; P = 0.11), pooled results were homogenous (P = 0.31; I^2^ = 4%). Figure 3C
4. **Suspected chorioamnionitis**
  Pooled studies [30, 36] measured numbers of pregnant women with suspected chorioamnionitis showed no significant difference between vaccinated and unvaccinated pregnant women (1.66% vs. 2.05%. RR 0.76; 95% CI 0.41 to 1.42; P = 0.39), pooled results were homogenous (P = 0.56; I^2^ = 0%) Figure 3D.

#### Maternal ICU admission

**Fig. 4:**
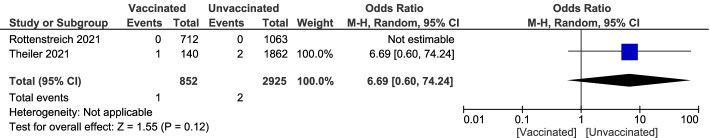
Forest plot of maternal ICU admission

Pooled studies [36, 38] recorded unassisted vaginal birth type in pregnant women showed no statistically significant difference between vaccinated and unvaccinated groups (58.6% vs. 65.2%. RR 6.69; 95% CI 0.60 to 74.24; *P* = 0.12). Figure 4

#### Obstetric outcomes

##### Miscarriage

**Fig. 5:**
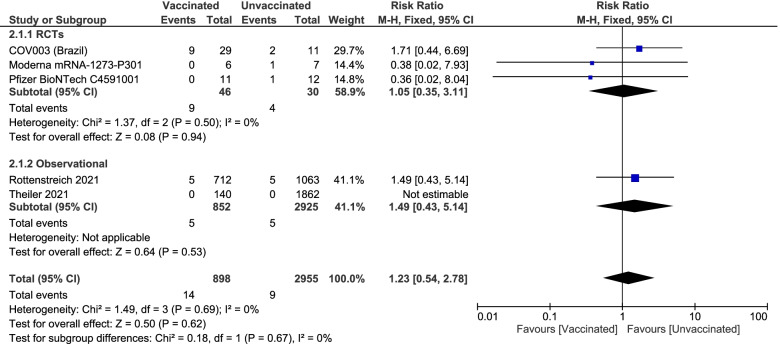
Forest plot of miscarriage

Pooled studies [28, 29, 34, 36, 38] showed no statistically significant difference in the incidence of miscarriage between vaccinated pregnant women and unvaccinated pregnant women (1.56% vs. 0.3%. RR 1.23; 95% CI 0.54 to 2.78; P = 0.62), pooled results were homogenous (P = 0.69; I^2^ = 0%). For the subgroup analysis, in the RCTs [28, 29, 34], the analysis showed no significant differences between the two groups (19.56% vs 13.33%. RR 1.05; 95% CI [0.35, 3.11]; P = 0.94), and the results were homogenous (P = 0.5; I.^2^ = 0%). For the observational studies [36, 38], there were no significant differences (0.59% vs. 0.17%. RR 1.49; 95% CI [0.43, 5.14]; P = 0.53). Figure 5

##### Birth type

**Fig. 6:**
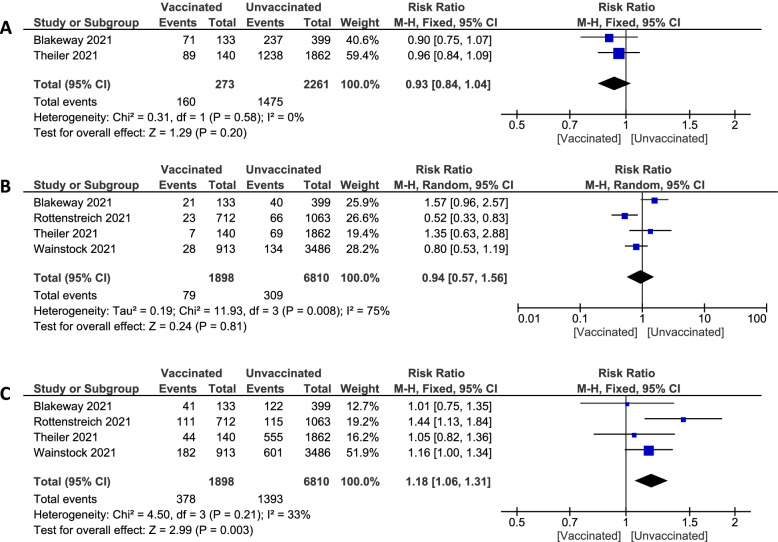
Forest plot of birth type; (**A**) unassisted vaginal delivery, (**B**) instrumental or vacuum-assisted, (**C**) cesarean

1. **Unassisted vaginal**
  Pooled studies [30, 38] recorded unassisted vaginal birth type in pregnant women showed no statistically significant difference between vaccinated and unvaccinated groups (58.6% vs. 65.2%. RR 0.93; 95% CI 0.84 to 1.04; P = 0.20), pooled results were homogenous (P = 0.58; I^2^ = 0%). Figure 6A
2. **Instrumental OR Vacuum-assisted delivery**
  Pooled studies [30, 36, 38, 39] measured birth type in a pregnant woman with either instrumental or vacuum-assisted delivery. They established no statistically significant difference between the vaccinated and unvaccinated groups (4.16% vs. 4.54%. RR 0.94; 95% CI 0.57 to 1.56; P = 0.81). Pooled results were heterogeneous, and the detected heterogeneity couldn't be solved (P = 0.008; I^2^ = 75%). Figure 6B
3. **Cesarean**
  Pooled studies [30, 36, 38, 39] showed a significant statistical difference which is associated with lower incidence of the cesarean section in the vaccinated group (19.92% vs 20.46%. RR 1.18; 95% CI 1.06 to 1.31; P = 0.003), pooled results were homogenous (P = 0.21; I^2^ = 33%). Figure 6C

##### Gestational age at delivery (week)

**Fig. 7:**
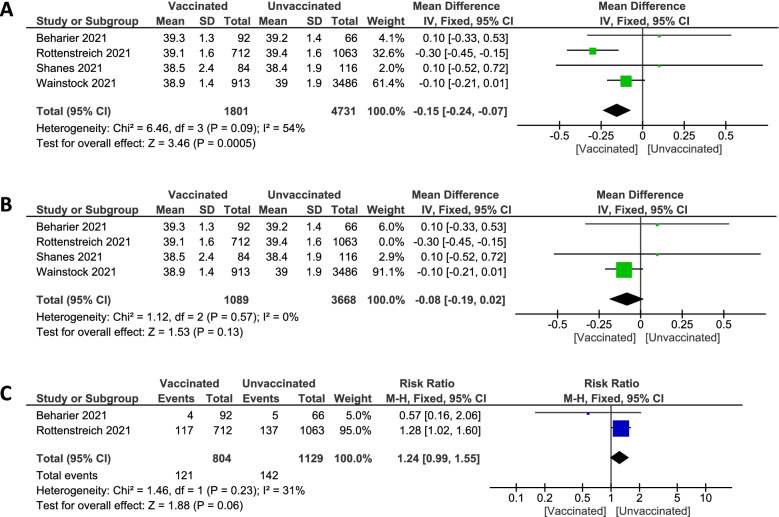
Forest plot of gestational age at delivery (week); (**A**) Before sensitivity analysis, (**B**) After sensitivity analysis, (**C**) Preterm birth

Pooled studies [20, 36, 37, 39] showed statistically significant reduction regarding gestational age at delivery in vaccinated pregnant women (MD -0.15; 95%CI -0.24 to -0.07; P = 0.0005), pooled results were heterogeneous (P = 0.09; I^2^ = 54%). Figure 7A The heterogeneity was solved by the exclusion of Rottenstreich et al. [36] after the random effect couldn't solve it (MD -0.08; 95%CI -0.19 to 0.02; P = 0.13), pooled results were homogenous (P = 0.57; I^2^ = 0%). Figure 7B

##### Preterm birth

Pooled studies [20, 36] recorded unassisted vaginal birth type in pregnant women showed no statistically significant difference between vaccinated and unvaccinated groups (15.0% vs. 12.6%. RR 1.24; 95% CI 0.99 to 1.55; P = 0.06), pooled results were homogenous (P = 0.23; I^2^ = 31%). Figure 7C

##### Neonates' outcomes

**Fig. 8:**
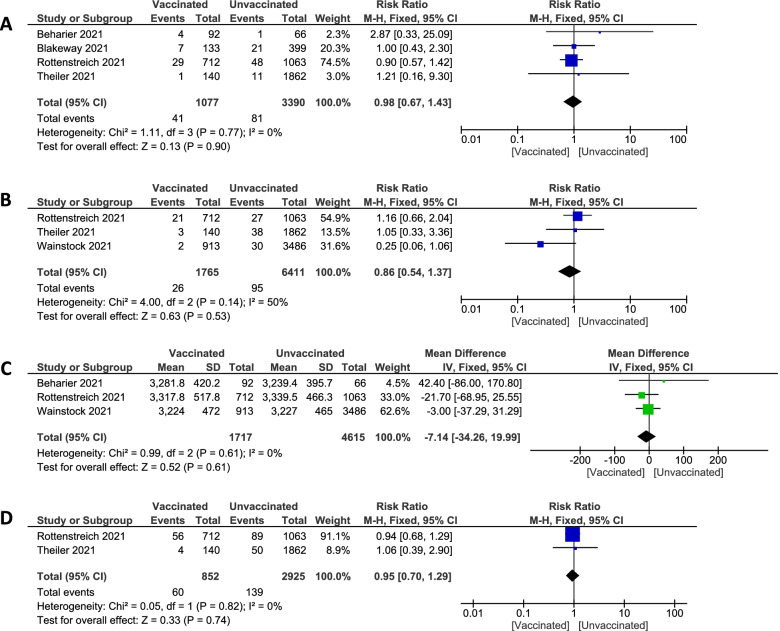
Forest plot of Neonates' outcomes; (**A**) neonatal unit admission, (**B**) Apgar ≤ 7 at 5 min, (**C**) Birthweight (gram), (**D**) Composite adverse neonatal outcome

1. **Neonatal unit admission**
  Pooled studies [20, 30, 36, 38] established no statistically significant difference between vaccinated and unvaccinated pregnant groups regarding numbers of admission to neonatal units (3.81% vs. 2.39%. RR 0.98; 95% CI 0.67 to 1.43; P = 0.90), pooled results were homogenous (P = 0.77; I^2^ = 0%). Figure 8A
2. **Apgar ≤ 7 at 5 min**
  Pooled studies [36, 38, 39] recorded the incidence of Apgar score ≤ 7 at 5 min revealed no statistically significant difference between vaccinated and unvaccinated groups (1.47% vs. 1.48%. RR 0.86; 95% CI 0.54 to 1.37; P = 0.53), pooled results were homogenous (P = 0.14; I^2^ = 50%). Figure 8B
3. **Birth Weight (gram)**
  Pooled studies [20, 36, 39] measured birthweight in the vaccinated pregnant women and unvaccinated women, and they found no statistically significant difference (MD -7.14; 95%CI -34.26 to 19.99; *P* = 0.61), pooled results were homogenous (*P* = 0.61; I^2^ = 0%). Figure 8C
4. **Composite adverse neonatal outcome**
  Pooled studies [36, 38] measured composite adverse neonatal outcomes in the vaccinated pregnant women and unvaccinated women, and they found no statistically significant difference (7.04% vs. 4.08%. RR 0.95; 95% CI 0.70 to 1.29; *P* = 0.74), pooled results were homogenous (*P* = 0.82; I^2^ = 0%). Figure 8D

## Discussion

In this systematic review meta-analysis, we focused on analyzing the safety of the COVID-19 vaccine regarding maternal, obstetric, and neonate outcomes. Almost all pregnant women are concerned about getting infected with SARS-CoV-2. However, they are far more concerned about vaccination due to the limited number of research investigating the safety of immunization against COVID-19 during pregnancy.

The risk of SARS-CoV-2 infection is not increased by pregnancy and labor[16]. Nevertheless, when comparing pregnant women of the same age to non-pregnant women of the same age, the clinical manifestation of COVID-19 appears to be significantly worse[41]; however, the vast majority of infected pregnant recover without having to give birth. It seems that women diagnosed with COVID-19, particularly those who developed pneumonia, have a higher incidence of pregnancy complications birth before 37 weeks of pregnancy and probably cesarean delivery, which is most likely associated with severe maternal disease[42]. We found that vaccination against COVID-19 had no differences in the incidence of miscarriage between vaccinated and unvaccinated pregnant women, Rottenstreich et al. [36] showed that women who received two doses of vaccination had more miscarriages in the past. Nevertheless, they found no statistically significant difference between vaccinated and unvaccinated arms. Theiler et al. [38] recorded that no women had a miscarriage in both groups. Pfizer [28], Moderna [34], and COV003 (Brazil) [29] found no significant difference in the incidence of miscarriage which supports our results.

Due to the special circumstances of COVID-19, pregnant women do not want to spend a long time in the hospital. Nevertheless, our analysis showed no difference between vaccinated and unvaccinated pregnant women. Three studies; Rottenstreich et al. [36], Theiler et al. [38], and Wainstock et al. [39], measured maternal hospitalization per day and also found no significant differences.

We analyzed intrapartum & postpartum complications for safety and focused on four major complications: Puerperal fever, postpartum hemorrhage, Placental abruption, and suspected chorioamnionitis. There was no difference between vaccinated and unvaccinated pregnant women regarding all intrapartum and postpartum complications that we analyzed. Blakeway et al. [30] recorded our four complications regarding intrapartum and postpartum. They found no differences between vaccinated and unvaccinated. Wainstock et al. [34] evaluated puerperal fever, postpartum hemorrhage, and placental abruption. Their results showed no statistically significant differences between the two groups, either vaccinated or not. This could be referred to some of our included studies that they included only women who get vaccinated in the third trimester. Therefore, we are unable to make any conclusions about the pregnant women who were vaccinated earlier in their early stages of pregnancy.

Also, our results agreed with all of the included studies regarding intrapartum and postpartum complications that their incidence showed no differences between the two groups and that may be affected by the pandemic's indirect impacts, such as changes in the availability of healthcare facilities and the behavior of pregnant women.

Regarding instrumental or vacuumed birth type, we found no significant difference between vaccinated pregnant women and unvaccinated. Rottenstreich et al. [36] found a significant increase in vacuum-assisted delivery in unvaccinated pregnant women. This could be explained as a normal finding since we utilize vacuum-assisted delivery for various reasons; including maternal tiredness, a worrisome fetal heart rate trace, a lengthy second stage of labor, or a desire to speed up the second stage of labor. Wainstock et al. [39], Blakeway et al. [30], and Theiler et al. [38] supported our results, and they discovered no difference between the two arms.

Many studies fail to discriminate between natural and iatrogenic premature birth. As a result of the assumption that the care of severe maternal respiratory illness would be improved by delivery, many third-trimester patients are delivered by planned cesarean. However, this theory has not been validated. On the other hand, we found an increase in the number of pregnant women who had a cesarean delivery in the unvaccinated group. Rottenstreich et al. [36] supported our results, however, Wainstock et al. [39], Blakeway et al. [30], and Theiler et al. [38] established no significant difference between the two groups. Since this group has a greater rate of previous cesarean section, which is a risk factor for a second cesarean section, we must reveal that even though the results are statistically significant, it is not significant clinically. We need to do so more studies.

Maternal illnesses with COVID-19 result in congenital infections that can be transmitted vertically, In utero, intrapartum, and during the early postnatal period. These routes appear to occur in a small percentage of COVID-19 in the third trimester. Infection rates of COVID-19 are also lower compared to other bacteria that cause congenital infection. Moreover, In the early stages of pregnancy, it's difficult to know the prevalence of vertical transmission and the resulting risk to a baby's health, especially since there aren't many studies available [43]. We focused on the neonates' outcomes as; neonatal unit admission, Apgar score, birth weight, and composite adverse events. Regarding the incidence of neonatal unit admission, we found no statistically significant difference between two vaccinated pregnant women, and unvaccinated group, Beharier et al. [20], Blakeway et al. [30], Rottenstreich et al. [36], and Theiler et al. [38] supported our results and found no statistically significant difference between both groups. These results could be explained in certain cases, that the time between the second vaccine dosage and birth may have too short to detect negative results, so we cannot say for sure that the vaccine does not cause neonatal adverse effects.

Besides Apgar score, some studies measured the incidence of Apgar score ≤ 7 at five minutes. We analyzed these results and found no significant difference between vaccinated pregnant women and unvaccinated. Rottenstreich et al. [36], Wainstock et al. [39], and Theiler et al. [38] supported our results and established no significant difference between the two arms. Despite the good results of vaccinated pregnant women regarding neonatal outcomes, we must do more research on rare adverse effects to ensure that the vaccine is safe.

Our results showed that there is no significant difference between vaccinated and unvaccinated pregnant groups. Maybe this finding is a result of that most of the published studies included pregnant women who got vaccinated in the third trimester, or they didn't mention it. So we couldn't decide which was good, to get vaccinated either early in pregnancy or not. For that reason, we need to do additional research to look at the differences in uncommon adverse birth outcomes and results following early and late pregnancy vaccination.

Accordingly, COVID-19 vaccination could be harmless for pregnant women, especially in the third trimester, to avoid any possible rare adverse outcomes for neonates.

The most significant advantages of our study are as follows: 1- As far as we know, this is the first meta-analysis in which the generalizability of the findings has been enhanced. 2- In general, most of our outcomes were homogeneous, and we were able to solve most of the heterogeneity if found by random effect or by leaving one study out of the analysis. 3- Relatively large sample size.

However, we have some limitations: 1- This review is confined to the short-term effect and did not evaluate the long-term results for vaccine safety criteria, such as the preterm birth rates and congenital fetal anomalies. 2- We included different study designs because there are limited studies on this topic. 3- All RCTs had a conflict of interest regarding other biases, and they had not enough information about sequence generation or allocation concealment, which could affect our results.

RCTs on the effect of vaccination in pregnant women with larger sample sizes and longer follow-up durations are recommended. Also, more RCTs should be done to compare pregnant women in the different trimesters in terms of efficacy and safety outcomes. It is also recommended to focus on neonatal outcomes and rare adverse events from vaccination.

## Conclusion

According to studies published until now, our results showed that in the short-term, COVID-19 vaccination is well tolerated regarding maternal and obstetric adverse effects when pregnant women get vaccinated in the third trimester. Furthermore, it decreases the complications that could be happened from SARS-CoV-2 infection. However, it is unclear whether the vaccine itself could harm or not for neonates when pregnant women get vaccinated in the first trimester.

## Supplementary Information

**Additional file 1:** **Supplemental Figure 1.** The effect of Mido(L)-ATRA on the content of Annexin V+ cells. HL-60 cells were treated with 0.25 μM modistaurin (M(L)) and/or 0.1 μM ATRA for 6 d. HL-60Res and U937 cells were treated with 0.1 μM modistaurin (M(L)) and/or 1 μM ATRA for 12 and 8 d, respectively. (A) The column graph of the content of Annexin V+ cells in three cell lines. Each value represents the mean ± SD of three independent measurements. (B) Representative scattered plotgrams of Annexin V expression. Results were representative among three independent experiments. **Supplemental Figure 2.** The effect of Mido(H)-ATRA on the content of CD11b+ cells. Cells were treated with 0.5 μM midostaurin (M(H)) and/or ATRA for 2 d. (A) The column graph of CD11b expression in three cell lines. Each value represents the mean ± SD of three independent measurements. ***P<0.005, versus DMSO-treated cells. (B) Representative histograms of CD11b expression with high dose midostaurin and/or ATRA. Results were representative among three independent experiments. Supplemental Figure 3. Most membranes were cut prior to hybridization. Original blots of the immunoblot detection shown in Fig 2A-Fig 2B, Fig 3D, Fig 4A-Fig 4C, Fig 5A and Fig 5E.

## Data Availability

The data that support the findings of this study are available upon reasonable request.

## References

[1] Wang C, Tee M, Roy AE, Fardin MA, Srichokchatchawan W, Habib HA (2021). The impact of COVID-19 pandemic on physical and mental health of Asians: A study of seven middle-income countries in Asia. PLoS ONE.

[2] Coronavirus W. Dashboard| WHO Coronavirus (COVID-19) Dashboard with vaccination data 2021 [Available from: https://covid19.who.int/.

[3] Edwards B, Biddle N, Gray M, Sollis K (2021). COVID-19 vaccine hesitancy and resistance: Correlates in a nationally representative longitudinal survey of the Australian population. PLoS ONE.

[4] Haas EJ, Angulo FJ, McLaughlin JM, Anis E, Singer SR, Khan F (2021). Impact and effectiveness of mRNA BNT162b2 vaccine against SARS-CoV-2 infections and COVID-19 cases, hospitalisations, and deaths following a nationwide vaccination campaign in Israel: an observational study using national surveillance data. Lancet.

[5] Vasileiou E, Simpson CR, Shi T, Kerr S, Agrawal U, Akbari A (2021). Interim findings from first-dose mass COVID-19 vaccination roll-out and COVID-19 hospital admissions in Scotland: a national prospective cohort study. Lancet.

[6] Dashraath P, Wong JLJ, Lim MXK, Lim LM, Li S, Biswas A (2020). Coronavirus disease 2019 (COVID-19) pandemic and pregnancy. Am J Obstet Gynecol.

[7] DeBolt CA, Bianco A, Limaye MA, Silverstein J, Penfield CA, Roman AS (2021). Pregnant women with severe or critical coronavirus disease 2019 have increased composite morbidity compared with nonpregnant matched controls. Am J Obstet Gynecol.

[8] Delahoy MJ, Whitaker M, Chai SJ, Daily Kirley P, Alden N, Kawasaki B, et al. Morbidity and Mortality Weekly Report Characteristics and Maternal and Birth Outcomes of Hospitalized Pregnant Women with Laboratory-Confirmed COVID-19-COVID-NET, 13 States. 2020. 10.15585/mmwr.mm6938e1 PMC7727497 32970655

[9] Juan J, Gil MM, Rong Z, Zhang Y, Yang H, Poon LC, Effect of coronavirus disease,  (2019). (COVID-19) on maternal, perinatal and neonatal outcome: systematic review. Ultrasound Obstet Gynecol.

[10] Knight M, Bunch K, Vousden N, Morris E, Simpson N, Gale C (2020). Characteristics and outcomes of pregnant women admitted to hospital with confirmed SARS-CoV-2 infection in UK: National population based cohort study. The BMJ.

[11] Perritt J, Grossman D (2021). The Health Consequences of Restrictive Abortion Laws. JAMA Intern Med.

[12] Pierce-Williams RAM, Burd J, Felder L, Khoury R, Bernstein PS, Avila K (2020). Clinical course of severe and critical coronavirus disease 2019 in hospitalized pregnancies: a United States cohort study. Am J Obstet Gynecol MFM.

[13] Zambrano LD, Ellington S, Strid P, Galang RR, Oduyebo T, Tong VT, et al. Update: Characteristics of Symptomatic Women of Reproductive Age with Laboratory-Confirmed SARS-CoV-2 Infection by Pregnancy Status — United States, January 22–October 3, 2020. 2020. 10.15585/mmwr.mm6944e3 PMC7643892 33151921

[14] COVID-19 Vaccination Considerations for Obstetric–Gynecologic Care | ACOG.

[15] Stafford IA, Parchem JG, Sibai BM (2021). The coronavirus disease 2019 vaccine in pregnancy: risks, benefits, and recommendations. Am J Obstet Gynecol.

[16] Allotey J, Stallings E, Bonet M, Yap M, Chatterjee S, Kew T, Clinical manifestations, risk factors, and maternal and perinatal outcomes of coronavirus disease,  (2019). in pregnancy: Living systematic review and meta-analysis. The BMJ.

[17] Hantoushzadeh S, Shamshirsaz AA, Aleyasin A, Seferovic MD, Aski SK, Arian SE (2020). Maternal death due to COVID-19. Am J Obstet Gynecol.

[18] LoMauro A, Aliverti A (2015). Respiratory physiology of pregnancy.

[19] Abu-Raya B, Madhi SA, Omer SB, Amirthalingam G, Giles ML, Flanagan KL, et al. Global Perspectives on Immunization Against SARS-CoV-2 During Pregnancy and Priorities for Future Research: An International Consensus Paper From the World Association of Infectious Diseases and Immunological Disorders. Frontiers in Immunology: Frontiers Media S.A.; 2021. 10.3389/fimmu.2021.808064 PMC8733958 35003137

[20] Beharier O, Mayo RP, Raz T, Sacks KN, Schreiber L, Suissa-Cohen Y (2021). Efficient maternal to neonatal transfer of antibodies against SARS-CoV-2 and BNT162b2 mRNA COVID-19 vaccine. J Clin Invest.

[21] Mithal Msci LB, Otero SBA, Shanes ED, Goldstein JA, Miller Mph ES. Cord blood antibodies following maternal coronavirus disease 2019 vaccination during pregnancy. 2021. 10.1016/j.ajog.2021.03.035 PMC8012273 33812808

[22] Rasmussen SA, Kelley CF, Horton JP, Jamieson DJ (2021). Coronavirus Disease 2019 (COVID-19) Vaccines and Pregnancy: What Obstetricians Need to Know. Obstet Gynecol.

[23] Skjefte M, Ngirbabul M, Akeju O, Escudero D, Hernandez-Diaz S, Wyszynski DF (2021). COVID-19 vaccine acceptance among pregnant women and mothers of young children: results of a survey in 16 countries. Eur J Epidemiol.

[24] Higgins JPT, Green S, Cochrane C. Cochrane handbook for systematic reviews of interventions: Wiley-Blackwell; 2008. 649- p.

[25] Page MJ, McKenzie JE, Bossuyt PM, Boutron I, Hoffmann TC, Mulrow CD (2021). The PRISMA 2020 statement: An updated guideline for reporting systematic reviews.

[26] Wells GA, Wells G, Shea B, Shea B, O'Connell D, Peterson J, et al., editors. The Newcastle-Ottawa Scale (NOS) for Assessing the Quality of Nonrandomised Studies in Meta-Analyses2014.

[27] Mustafa RA, Santesso N, Brozek J, Akl EA, Walter SD, Norman G (2013). The GRADE approach is reproducible in assessing the quality of evidence of quantitative evidence syntheses. J Clin Epidemiol.

[28] A PHASE 1/2/3, PLACEBO-CONTROLLED Title: A Phase 1/2/3 Study to Evaluate the Safety, Tolerability, Immunogenicity, and Efficacy of RNA Vaccine Candidates Against COVID-19 in Healthy Individuals.

[29] A Study of a Candidate COVID-19 Vaccine (COV003) - Full Text View - ClinicalTrials.gov.

[30] Blakeway H, Prasad S, Kalafat E, Heath PT, Ladhani SN, Le Doare K (2022). COVID-19 vaccination during pregnancy: coverage and safety. Am J Obstet Gynecol.

[31] Butt A, Abou-Samra A-B, Al Khal A, Coyle P, Saleh H, Kaleeckal AH, et al. Effectiveness of the SARS-CoV-2 mRNA Vaccines in Pregnant Women. 10.1172/JCI153662 PMC8631593 34618693

[32] Collier ARY, McMahan K, Yu J, Tostanoski LH, Aguayo R, Ansel J (2021). Immunogenicity of COVID-19 mRNA Vaccines in Pregnant and Lactating Women. JAMA - Journal of the American Medical Association.

[33] Dagan N, Barda N, Biron-Shental T, Makov-Assif M, Key C, Kohane IS (2021). Effectiveness of the BNT162b2 mRNA COVID-19 vaccine in pregnancy. Nat Med.

[34] Fda, Cber. Vaccines and Related Biological Products Advisory Committee December 17, 2020 Meeting Briefing Document - FDA. 2020.

[35] Kharbanda EO, Haapala J, DeSilva M, Vazquez-Benitez G, Vesco KK, Naleway AL (2021). Spontaneous Abortion Following COVID-19 Vaccination During Pregnancy. JAMA.

[36] Rottenstreich M, Sela HY, Rotem R, Kadish E, Wiener-Well Y, Grisaru-Granovsky S (2022). Covid-19 vaccination during the third trimester of pregnancy: rate of vaccination and maternal and neonatal outcomes, a multicentre retrospective cohort study. BJOG.

[37] Shanes ED, Otero S, Mithal LB, Mupanomunda CA, Miller ES, Goldstein JA (2021). Severe Acute Respiratory Syndrome Coronavirus 2 (SARS-CoV-2) Vaccination in Pregnancy: Measures of Immunity and Placental Histopathology. Obstet Gynecol.

[38] Theiler RN, Wick M, Mehta R, Weaver AL, Virk A, Swift M (2021). Pregnancy and birth outcomes after SARS-CoV-2 vaccination in pregnancy. Am J Obstet Gynecol MFM..

[39] Wainstock T, Yoles I, Sergienko R, Sheiner E (2021). Prenatal maternal COVID-19 vaccination and pregnancy outcomes. Vaccine.

[40] Tracking SARS-CoV-2 variants: WHO; 2022 [updated 25 May 2022. Available from: https://www.who.int/activities/tracking-SARS-CoV-2-variants.

[41] Lokken EM, Huebner EM, Taylor GG, Hendrickson S, Vanderhoeven J, Kachikis A (2021). Disease severity, pregnancy outcomes, and maternal deaths among pregnant patients with severe acute respiratory syndrome coronavirus 2 infection in Washington State. Am J Obstet Gynecol.

[42] Metz TD, Clifton RG, Hughes BL, Sandoval G, Saade GR, Grobman WA (2021). Disease Severity and Perinatal Outcomes of Pregnant Patients With Coronavirus Disease 2019 (COVID-19). Obstet Gynecol.

[43] Kotlyar AM, Grechukhina O, Chen A, Popkhadze S, Grimshaw A, Tal O (2021). Vertical transmission of coronavirus disease 2019: a systematic review and meta-analysis. Am J Obstet Gynecol.

