# Supplementary material for: Maternal and neonatal safety outcomes after SAR-CoV-2 vaccination during pregnancy: a systematic review and meta-analysis
Source: BMC Pregnancy Childbirth. 2022 Jul 21;22:581. doi: 10.1186/s12884-022-04884-9 (PMC9302221; doi:10.1186/s12884-022-04884-9)
Supplement: Supplementary file 1 — Additional file 1: Supplemental Figure 1. The effect of Mido(L)-ATRA on the content of Annexin V+ cells. HL-60 cells were treated with 0.25 μM modistaurin (M(L)) and/or 0.1 μM ATRA for 6 d. HL-60Res and U937 cells were treated with 0.1 μM modistaurin (M(L)) and/or 1 μM ATRA for 12 and 8 d, respectively. (A) The column graph of the content of Annexin V+ cells in three cell lines. Each value represents the mean ± SD of three independent measurements. (B) Representative scattered plotgrams of Annexin V expression. Results were representative among three independent experiments. Supplemental Figure 2. The effect of Mido(H)-ATRA on the content of CD11b+ cells. Cells were treated with 0.5 μM midostaurin (M(H)) and/or ATRA for 2 d. (A) The column graph of CD11b expression in three cell lines. Each value represents the mean ± SD of three independent measurements. ***P<0.005, versus DMSO-treated cells. (B) Representative histograms of CD11b expression with high dose midostaurin and/or ATRA. Results were representative among three independent experiments. Supplemental Figure 3. Most membranes were cut prior to hybridization. Original blots of the immunoblot detection shown in Fig 2A-Fig 2B, Fig 3D, Fig 4A-Fig 4C, Fig 5A and Fig 5E. [file 12884_2022_4884_MOESM1_ESM.zip › Supplementary File 1.docx]

The search strategy was implemented in the current review.

| **PubMed query** | | **Results** |
| --- | --- | --- |
| 1 | (Gestation OR Pregnan*) | 1,092,878 |
| 2 | (COVID 19 Vaccine OR COVID-19 Vaccine OR COVID19 Vaccine OR COVID-19 Virus Vaccine OR COVID 19 Virus Vaccine OR COVID19 Virus Vaccine OR SARS-CoV-2 Vaccine OR SARS CoV 2 Vaccine OR SARS2 Vaccine OR Coronavirus Disease 2019 Vaccine OR Coronavirus Disease 2019 Virus Vaccine OR Coronavirus Disease-19 Vaccine OR Coronavirus Disease 19 Vaccine OR 2019-nCoV Vaccine OR 2019 nCoV Vaccine OR 2019 Novel Coronavirus Vaccine OR 2019-nCoV Vaccine OR 2019 nCoV Vaccine OR SARS Coronavirus 2 Vaccines OR "COVID-19 Vaccines"[Mesh]) | 28,938 |
| 3 | #1 AND #2 | 695 |
| **Scopus query** | | **Results** |
| (Gestation OR Pregnan*) AND ("COVID 19 Vaccine" OR "COVID-19 Vaccine" OR "COVID19 Vaccine" OR "COVID-19 Virus Vaccine" OR "COVID 19 Virus Vaccine" OR "COVID19 Virus Vaccine" OR "SARS-CoV-2 Vaccine" OR "SARS CoV 2 Vaccine" OR "SARS2 Vaccine" OR "Coronavirus Disease 2019 Vaccine" OR "Coronavirus Disease 2019 Virus Vaccine" OR "Coronavirus Disease-19 Vaccine" OR "Coronavirus Disease 19 Vaccine" OR "2019-nCoV Vaccine" OR "2019 nCoV Vaccine" OR "2019 Novel Coronavirus Vaccine" OR "2019-nCoV Vaccine" OR "2019 nCoV Vaccine" OR "SARS Coronavirus 2 Vaccines") | | 666 |
| **Web of Science query** | | **Results** |
| (Gestation OR Pregnan*) AND (COVID 19 Vaccine OR COVID-19 Vaccine OR COVID19 Vaccine OR COVID-19 Virus Vaccine OR COVID 19 Virus Vaccine OR COVID19 Virus Vaccine OR SARS-CoV-2 Vaccine OR SARS CoV 2 Vaccine OR SARS2 Vaccine OR Coronavirus Disease 2019 Vaccine OR Coronavirus Disease 2019 Virus Vaccine OR Coronavirus Disease-19 Vaccine OR Coronavirus Disease 19 Vaccine OR 2019-nCoV Vaccine OR 2019 nCoV Vaccine OR 2019 Novel Coronavirus Vaccine OR 2019-nCoV Vaccine OR 2019 nCoV Vaccine OR SARS Coronavirus 2 Vaccines) | | 212 |
| **Cochrane library query** | | **Results** |
| (Gestation OR Pregnan*) AND (COVID 19 Vaccine OR COVID-19 Vaccine OR COVID19 Vaccine OR COVID-19 Virus Vaccine OR COVID 19 Virus Vaccine OR COVID19 Virus Vaccine OR SARS-CoV-2 Vaccine OR SARS CoV 2 Vaccine OR SARS2 Vaccine OR Coronavirus Disease 2019 Vaccine OR Coronavirus Disease 2019 Virus Vaccine OR Coronavirus Disease-19 Vaccine OR Coronavirus Disease 19 Vaccine OR 2019-nCoV Vaccine OR 2019 nCoV Vaccine OR 2019 Novel Coronavirus Vaccine OR 2019-nCoV Vaccine OR 2019 nCoV Vaccine OR SARS Coronavirus 2 Vaccines | | 65 |
| **Embase** | | **Results** |
| (Gestation OR Pregnan*) AND (COVID 19 Vaccine OR COVID-19 Vaccine OR COVID19 Vaccine OR COVID-19 Virus Vaccine OR COVID 19 Virus Vaccine OR COVID19 Virus Vaccine OR SARS-CoV-2 Vaccine OR SARS CoV 2 Vaccine OR SARS2 Vaccine OR Coronavirus Disease 2019 Vaccine OR Coronavirus Disease 2019 Virus Vaccine OR Coronavirus Disease-19 Vaccine OR Coronavirus Disease 19 Vaccine OR 2019-nCoV Vaccine OR 2019 nCoV Vaccine OR 2019 Novel Coronavirus Vaccine OR 2019-nCoV Vaccine OR 2019 nCoV Vaccine OR SARS Coronavirus 2 Vaccines) | | 1017 |
| **OVID** | | **Results** |
| (Gestation OR Pregnan*) AND (COVID 19 Vaccine OR COVID-19 Vaccine OR COVID19 Vaccine OR COVID-19 Virus Vaccine OR COVID 19 Virus Vaccine OR COVID19 Virus Vaccine OR SARS-CoV-2 Vaccine OR SARS CoV 2 Vaccine OR SARS2 Vaccine OR Coronavirus Disease 2019 Vaccine OR Coronavirus Disease 2019 Virus Vaccine OR Coronavirus Disease-19 Vaccine OR Coronavirus Disease 19 Vaccine OR 2019-nCoV Vaccine OR 2019 nCoV Vaccine OR 2019 Novel Coronavirus Vaccine OR 2019-nCoV Vaccine OR 2019 nCoV Vaccine OR SARS Coronavirus 2 Vaccines) | | 673 |
| **medRxiv and bioRxiv** | | **Results** |
| topic1 <- c("vaccine","vaccines","vaccination","vaccinations")  topic2 <- c("pregnancy","pregnant"," Gestation ")  topic3 <- c("COVID","COVID-19","SARS-CoV-2") | | 227 |
| **Clinical trial.gov** | | **Results** |
| COVID-19 OR COVID-19 vaccine | | 250 |
| **Research Square** | | **Results** |
| Pregnancy AND vaccine | | 1376 |
| **Open gray** | | **Results** |
| (Gestation OR Pregnan*) AND (COVID 19 Vaccine OR COVID-19 Vaccine OR COVID19 Vaccine OR COVID-19 Virus Vaccine OR COVID 19 Virus Vaccine OR COVID19 Virus Vaccine OR SARS-CoV-2 Vaccine OR SARS CoV 2 Vaccine OR SARS2 Vaccine OR Coronavirus Disease 2019 Vaccine OR Coronavirus Disease 2019 Virus Vaccine OR Coronavirus Disease-19 Vaccine OR Coronavirus Disease 19 Vaccine OR 2019-nCoV Vaccine OR 2019 nCoV Vaccine OR 2019 Novel Coronavirus Vaccine OR 2019-nCoV Vaccine OR 2019 nCoV Vaccine OR SARS Coronavirus 2 Vaccines) | | 247 |
| **Gray literature** | | **Results** |
| (Gestation OR Pregnan*) AND (COVID 19 Vaccine OR COVID-19 Vaccine OR COVID19 Vaccine OR COVID-19 Virus Vaccine OR COVID 19 Virus Vaccine OR COVID19 Virus Vaccine OR SARS-CoV-2 Vaccine OR SARS CoV 2 Vaccine OR SARS2 Vaccine OR Coronavirus Disease 2019 Vaccine OR Coronavirus Disease 2019 Virus Vaccine OR Coronavirus Disease-19 Vaccine OR Coronavirus Disease 19 Vaccine OR 2019-nCoV Vaccine OR 2019 nCoV Vaccine OR 2019 Novel Coronavirus Vaccine OR 2019-nCoV Vaccine OR 2019 nCoV Vaccine OR SARS Coronavirus 2 Vaccines) | | 0 |
