# Supplementary material for: Maternal and neonatal safety outcomes after SAR-CoV-2 vaccination during pregnancy: a systematic review and meta-analysis
Source: BMC Pregnancy Childbirth. 2022 Jul 21;22:581. doi: 10.1186/s12884-022-04884-9 (PMC9302221; doi:10.1186/s12884-022-04884-9)
Supplement: Supplementary file 1 — Additional file 1: Supplemental Figure 1. The effect of Mido(L)-ATRA on the content of Annexin V+ cells. HL-60 cells were treated with 0.25 μM modistaurin (M(L)) and/or 0.1 μM ATRA for 6 d. HL-60Res and U937 cells were treated with 0.1 μM modistaurin (M(L)) and/or 1 μM ATRA for 12 and 8 d, respectively. (A) The column graph of the content of Annexin V+ cells in three cell lines. Each value represents the mean ± SD of three independent measurements. (B) Representative scattered plotgrams of Annexin V expression. Results were representative among three independent experiments. Supplemental Figure 2. The effect of Mido(H)-ATRA on the content of CD11b+ cells. Cells were treated with 0.5 μM midostaurin (M(H)) and/or ATRA for 2 d. (A) The column graph of CD11b expression in three cell lines. Each value represents the mean ± SD of three independent measurements. ***P<0.005, versus DMSO-treated cells. (B) Representative histograms of CD11b expression with high dose midostaurin and/or ATRA. Results were representative among three independent experiments. Supplemental Figure 3. Most membranes were cut prior to hybridization. Original blots of the immunoblot detection shown in Fig 2A-Fig 2B, Fig 3D, Fig 4A-Fig 4C, Fig 5A and Fig 5E. [file 12884_2022_4884_MOESM1_ESM.zip › Supplementary File 2.docx]

The reasons for excluding articles after reading the full text.

| **Cause** | **Studies** |
| --- | --- |
| **Ineligible population**   1. Studies excluded pregnant females (n = 9) 2. Breastfeeding or lactating women (n = 12) 3. Studies included infected females not vaccinated (n = 4) 4. One group or more is non-pregnant women (n = 4) | (1-29) |
| **Wrong control**   1. No unvaccinated group | (30-47) |
| **Wrong outcome**   1. Studies reported Fertility as the outcome of interest (n = 3) 2. Studies reported Attitude and Acceptance as the outcome of interest (n = 10) | (48-60) |
| **Wrong study type**   1. Erratum n = )6) 2. Note (n = 18) 3. Letter (n = 16) 4. Case report (n = 4) 5. Review (n = 100) 6. Editorial (n = 34) 7. Guidelines (n = 1) 8. Short survey (n = 9) 9. Audio interview (n = 1) 10. Correction (n = 1) | (61-238) |
| Animal studies | (239-252) |
| Studies omitted for a variety of reasons | (253-263) |

**References**

1. Akova M, Unal S. A randomized, double-blind, placebo-controlled phase III clinical trial to evaluate the efficacy and safety of SARS-CoV-2 vaccine (inactivated, Vero cell): a structured summary of a study protocol for a randomised controlled trial. Trials. 2021;22(1):276.

2. Zipursky JS, Greenberg RA, Maxwell C, Bogler T. Pregnancy, breastfeeding and the SARS-CoV-2 vaccine: an ethics-based framework for shared decision-making. Cmaj. 2021;193(9):E312-e4.

3. Valcarce V, Stafford LS, Neu J, Cacho N, Parker L, Mueller M, et al. Detection of SARS-CoV-2-Specific IgA in the Human Milk of COVID-19 Vaccinated Lactating Health Care Workers. Breastfeed Med. 2021;16(12):1004-9.

4. Rosenberg-Friedman M, Kigel A, Bahar Y, Werbner M, Alter J, Yogev Y, et al. BNT162b2 mRNA vaccine elicited antibody response in blood and milk of breastfeeding women. Nat Commun. 2021;12(1):6222.

5. Baird JK, Jensen SM, Urba WJ, Fox BA, Baird JR. SARS-CoV-2 Antibodies Detected in Mother's Milk Post-Vaccination. J Hum Lact. 2021;37(3):492-8.

6. Charepe N, Gonçalves J, Juliano AM, Lopes DG, Canhão H, Soares H, et al. COVID-19 mRNA vaccine and antibody response in lactating women: a prospective cohort study. BMC Pregnancy Childbirth. 2021;21(1):632.

7. Esteve-Palau E, Gonzalez-Cuevas A, Guerrero ME, Garcia-Terol C, Alvarez MC, Casadevall D, et al. Quantification of Specific Antibodies Against SARS-CoV-2 in Breast Milk of Lactating Women Vaccinated With an mRNA Vaccine. JAMA Netw Open. 2021;4(8):e2120575.

8. Golan Y, Prahl M, Cassidy AG, Gay C, Wu AHB, Jigmeddagva U, et al. COVID-19 mRNA Vaccination in Lactation: Assessment of adverse events and vaccine related antibodies in mother-infant dyads. medRxiv. 2021.

9. Guida M, Terracciano D, Cennamo M, Aiello F, La Civita E, Esposito G, et al. COVID-19 Vaccine mRNABNT162b2 Elicits Human Antibody Response in Milk of Breastfeeding Women. Vaccines (Basel). 2021;9(7).

10. Juncker HG, Romijn M, Loth VN, Ruhé EJM, Bakker S, Kleinendorst S, et al. Antibodies Against SARS-CoV-2 in Human Milk: Milk Conversion Rates in the Netherlands. J Hum Lact. 2021;37(3):469-76.

11. McLaurin-Jiang S, Garner CD, Krutsch K, Hale TW. Maternal and Child Symptoms Following COVID-19 Vaccination Among Breastfeeding Mothers. Breastfeed Med. 2021;16(9):702-9.

12. Perl SH, Uzan-Yulzari A, Klainer H, Asiskovich L, Youngster M, Rinott E, et al. SARS-CoV-2-Specific Antibodies in Breast Milk After COVID-19 Vaccination of Breastfeeding Women. Jama. 2021;325(19):2013-4.

13. Schwartz A, Nir O, Toussia-Cohen S, Leibovich L, Strauss T, Asraf K, et al. Presence of SARS-CoV-2 antibodies in lactating women and their infants following BNT162b2 messenger RNA vaccine. Am J Obstet Gynecol. 2021;225(5):577-9.

14. Golan Y, Prahl M, Cassidy A, Lin CY, Ahituv N, Flaherman VJ, et al. Evaluation of Messenger RNA From COVID-19 BTN162b2 and mRNA-1273 Vaccines in Human Milk. JAMA Pediatr. 2021;175(10):1069-71.

15. Rathberger K, Häusler S, Wellmann S, Weigl M, Langhammer F, Bazzano MV, et al. SARS-CoV-2 in pregnancy and possible transfer of immunity: assessment of peripartal maternal and neonatal antibody levels and a longitudinal follow-up. J Perinat Med. 2021;49(6):702-8.

16. Wu T, Zuo Z, Yang D, Luo X, Jiang L, Xia Z, et al. Venous thromboembolic events in patients with COVID-19: a systematic review and meta-analysis. Age Ageing. 2021;50(2):284-93.

17. Chang WH. A review of vaccine effects on women in light of the COVID-19 pandemic. Taiwan J Obstet Gynecol. 2020;59(6):812-20.

18. Pullen KM, Atyeo C, Collier AY, Gray KJ, Belfort MB, Lauffenburger DA, et al. Selective functional antibody transfer into the breastmilk after SARS-CoV-2 infection. Cell Rep. 2021;37(6):109959.

19. Timircan M, Bratosin F, Vidican I, Suciu O, Tirnea L, Avram V, et al. Exploring Pregnancy Outcomes Associated with SARS-CoV-2 Infection. Medicina (Kaunas). 2021;57(8).

20. Joseph NT, Dude CM, Verkerke HP, Irby LS, Dunlop AL, Patel RM, et al. Maternal Antibody Response, Neutralizing Potency, and Placental Antibody Transfer After Severe Acute Respiratory Syndrome Coronavirus 2 (SARS-CoV-2) Infection. Obstet Gynecol. 2021;138(2):189-97.

21. Maru S, Patil U, Carroll-Bennett R, Baum A, Bohn-Hemmerdinger T, Ditchik A, et al. Universal screening for SARS-CoV-2 infection among pregnant women at Elmhurst Hospital Center, Queens, New York. PLoS One. 2020;15(12):e0238409.

22. Xu S, Shao F, Bao B, Ma X, Xu Z, You J, et al. Clinical Manifestation and Neonatal Outcomes of Pregnant Patients With Coronavirus Disease 2019 Pneumonia in Wuhan, China. Open Forum Infect Dis. 2020;7(7):ofaa283.

23. Baden LR, El Sahly HM, Essink B, Kotloff K, Frey S, Novak R, et al. Efficacy and Safety of the mRNA-1273 SARS-CoV-2 Vaccine. N Engl J Med. 2021;384(5):403-16.

24. Atyeo C, DeRiso EA, Davis C, Bordt EA, De Guzman RM, Shook LL, et al. COVID-19 mRNA vaccines drive differential antibody Fc-functional profiles in pregnant, lactating, and nonpregnant women. Sci Transl Med. 2021;13(617):eabi8631.

25. Atyeo C, DeRiso EA, Davis C, Bordt EA, DeGuzman RM, Shook LL, et al. COVID-19 mRNA vaccines drive differential Fc-functional profiles in pregnant, lactating, and non-pregnant women. bioRxiv. 2021.

26. Geisen UM, Berner DK, Tran F, Sümbül M, Vullriede L, Ciripoi M, et al. Immunogenicity and safety of anti-SARS-CoV-2 mRNA vaccines in patients with chronic inflammatory conditions and immunosuppressive therapy in a monocentric cohort. Ann Rheum Dis. 2021;80(10):1306-11.

27. Gray KJ, Bordt EA, Atyeo C, Deriso E, Akinwunmi B, Young N, et al. COVID-19 vaccine response in pregnant and lactating women: a cohort study. medRxiv. 2021.

28. Hirshberg JS, Huysman BC, Oakes MC, Cater EB, Odibo AO, Raghuraman N, et al. Offering onsite COVID-19 vaccination to high-risk obstetrical patients: initial findings. Am J Obstet Gynecol MFM. 2021;3(6):100478.

29. Behmard V, Bahri N, Mohammadzadeh F, Noghabi AD, Bahri N. Relationships between anxiety induced by COVID-19 and perceived social support among Iranian pregnant women. J Psychosom Obstet Gynaecol. 2021:1-8.

30. Zauche LH, Wallace B, Smoots AN, Olson CK, Oduyebo T, Kim SY, et al. Receipt of mRNA COVID-19 vaccines preconception and during pregnancy and risk of self-reported spontaneous abortions, CDC v-safe COVID-19 Vaccine Pregnancy Registry 2020-21. Res Sq. 2021.

31. Stuckelberger S, Favre G, Ceulemans M, Nordeng H, Gerbier E, Lambelet V, et al. SARS-CoV-2 Vaccine Willingness among Pregnant and Breastfeeding Women during the First Pandemic Wave: A Cross-Sectional Study in Switzerland. Viruses. 2021;13(7).

32. Rottenstreich A, Zarbiv G, Oiknine-Djian E, Zigron R, Wolf DG, Porat S. Efficient Maternofetal Transplacental Transfer of Anti- Severe Acute Respiratory Syndrome Coronavirus 2 (SARS-CoV-2) Spike Antibodies After Antenatal SARS-CoV-2 BNT162b2 Messenger RNA Vaccination. Clin Infect Dis. 2021;73(10):1909-12.

33. Riley LE. mRNA Covid-19 Vaccines in Pregnant Women. N Engl J Med. 2021;384(24):2342-3.

34. Prabhu M, Murphy EA, Sukhu AC, Yee J, Singh S, Eng D, et al. Antibody Response to Coronavirus Disease 2019 (COVID-19) Messenger RNA Vaccination in Pregnant Women and Transplacental Passage Into Cord Blood. Obstet Gynecol. 2021;138(2):278-80.

35. Kelly JC, Carter EB, Raghuraman N, Nolan LS, Gong Q, Lewis AN, et al. Anti-severe acute respiratory syndrome coronavirus 2 antibodies induced in breast milk after Pfizer-BioNTech/BNT162b2 vaccination. Am J Obstet Gynecol. 2021;225(1):101-3.

36. Kadali RAK, Janagama R, Peruru SR, Racherla S, Tirumala R, Madathala RR, et al. Adverse effects of COVID-19 messenger RNA vaccines among pregnant women: a cross-sectional study on healthcare workers with detailed self-reported symptoms. Am J Obstet Gynecol. 2021;225(4):458-60.

37. Kachikis A, Englund JA, Singleton M, Covelli I, Drake AL, Eckert LO. Short-term Reactions Among Pregnant and Lactating Individuals in the First Wave of the COVID-19 Vaccine Rollout. JAMA Netw Open. 2021;4(8):e2121310.

38. Ben-Mayor Bashi T, Amikam U, Ashwal E, Hershkovitz G, Attali E, Berkovitz-Shperling R, et al. The association of maternal SARS-CoV-2 vaccination-to-delivery interval and the levels of maternal and cord blood antibodies. Int J Gynaecol Obstet. 2022;156(3):436-43.

39. Zdanowski W, Waśniewski T. Evaluation of SARS-CoV-2 Spike Protein Antibody Titers in Cord Blood after COVID-19 Vaccination during Pregnancy in Polish Healthcare Workers: Preliminary Results. Vaccines (Basel). 2021;9(6).

40. Wang PH, Lee WL, Yang ST, Tsui KH, Chang CC, Lee FK. The impact of COVID-19 in pregnancy: Part II. Vaccination to pregnant women. J Chin Med Assoc. 2021;84(10):903-10.

41. Trostle ME, Limaye MA, Avtushka V, Lighter JL, Penfield CA, Roman AS. COVID-19 vaccination in pregnancy: early experience from a single institution. Am J Obstet Gynecol MFM. 2021;3(6):100464.

42. Shimabukuro TT, Kim SY, Myers TR, Moro PL, Oduyebo T, Panagiotakopoulos L, et al. Preliminary Findings of mRNA Covid-19 Vaccine Safety in Pregnant Persons. N Engl J Med. 2021;384(24):2273-82.

43. Sarwal Y, Sarwal T, Sarwal R. Vaccination of pregnant women against COVID-19 in India and Indonesia: Moving beyond the opt-in to the opt-out option. Int J Gynaecol Obstet. 2021;155(3):549-50.

44. Bookstein Peretz S, Regev N, Novick L, Nachshol M, Goffer E, Ben-David A, et al. Short-term outcome of pregnant women vaccinated with BNT162b2 mRNA COVID-19 vaccine. Ultrasound Obstet Gynecol. 2021;58(3):450-6.

45. Cassaniti I, Percivalle E, Zelini P, Ngaradoumbe Nanhorngue K, Parolo A, Bernardi V, et al. Both SARS-CoV-2 infection and vaccination in pregnancy elicited neutralizing antibodies in pregnant women and newborns. Clin Microbiol Infect. 2021;27(11):1708-9.

46. Fell DB, Dimitris MC, Hutcheon JA, Ortiz JR, Platt RW, Regan AK, et al. Guidance for design and analysis of observational studies of fetal and newborn outcomes following COVID-19 vaccination during pregnancy. Vaccine. 2021;39(14):1882-6.

47. Gray KJ. Maternal COVID-19 vaccine antibody response and passage into cord blood. J Pediatr. 2021;236:325-8.

48. Schaler L, Wingfield M. COVID-19 vaccine - can it affect fertility? Ir J Med Sci. 2021:1-3.

49. Schaal NK, Zöllkau J, Hepp P, Fehm T, Hagenbeck C. Pregnant and breastfeeding women's attitudes and fears regarding the COVID-19 vaccination. Arch Gynecol Obstet. 2021:1-8.

50. Iacobucci G. Covid-19: No evidence that vaccines can affect fertility, says new guidance. Bmj. 2021;372:n509.

51. Verma S, Carter EB, Mysorekar IU. SARS-CoV2 and pregnancy: An invisible enemy? Am J Reprod Immunol. 2020;84(5):e13308.

52. Tao L, Wang R, Han N, Liu J, Yuan C, Deng L, et al. Acceptance of a COVID-19 vaccine and associated factors among pregnant women in China: a multi-center cross-sectional study based on health belief model. Hum Vaccin Immunother. 2021;17(8):2378-88.

53. Sutton D, D'Alton M, Zhang Y, Kahe K, Cepin A, Goffman D, et al. COVID-19 vaccine acceptance among pregnant, breastfeeding, and nonpregnant reproductive-aged women. Am J Obstet Gynecol MFM. 2021;3(5):100403.

54. Skjefte M, Ngirbabul M, Akeju O, Escudero D, Hernandez-Diaz S, Wyszynski DF, et al. COVID-19 vaccine acceptance among pregnant women and mothers of young children: results of a survey in 16 countries. Eur J Epidemiol. 2021;36(2):197-211.

55. Shook LL, Kishkovich TP, Edlow AG. Countering COVID-19 Vaccine Hesitancy in Pregnancy: the "4 Cs". Am J Perinatol. 2021.

56. Mappa I, Luviso M, Distefano FA, Carbone L, Maruotti GM, Rizzo G. Women perception of SARS-CoV-2 vaccination during pregnancy and subsequent maternal anxiety: a prospective observational study. J Matern Fetal Neonatal Med. 2021:1-4.

57. Nguyen LH, Hoang MT, Nguyen LD, Ninh LT, Nguyen HTT, Nguyen AD, et al. Acceptance and willingness to pay for COVID-19 vaccines among pregnant women in Vietnam. Trop Med Int Health. 2021;26(10):1303-13.

58. Mose A, Yeshaneh A. COVID-19 Vaccine Acceptance and Its Associated Factors Among Pregnant Women Attending Antenatal Care Clinic in Southwest Ethiopia: Institutional-Based Cross-Sectional Study. Int J Gen Med. 2021;14:2385-95.

59. Xu B, Gao X, Zhang X, Hu Y, Yang H, Zhou YH. Real-World Acceptance of COVID-19 Vaccines among Healthcare Workers in Perinatal Medicine in China. Vaccines (Basel). 2021;9(7).

60. Shamshirsaz AA, Hessami K, Morain S, Afshar Y, Nassr AA, Arian SE, et al. Intention to Receive COVID-19 Vaccine during Pregnancy: A Systematic Review and Meta-analysis. Am J Perinatol. 2022;39(5):492-500.

61. Adhikari EH, Spong CY. COVID-19 Vaccination in Pregnant and Lactating Women. Jama. 2021;325(11):1039-40.

62. Beigi RH, Krubiner C, Jamieson DJ, Lyerly AD, Hughes B, Riley L, et al. The need for inclusion of pregnant women in COVID-19 vaccine trials. Vaccine. 2021;39(6):868-70.

63. Fesler MC, Stricker RB. Pre-Exposure Prophylaxis for COVID-19 in Pregnant Women. Int J Gen Med. 2021;14:279-84.

64. Lichert F. COVID-19 Vaccines also suitable for Pregnant Women? Geburtshilfe Und Frauenheilkunde. 2021:1-.

65. Maykin MM, Heuser C, Feltovich H. Pregnant people deserve the protection offered by SARS-CoV-2 vaccines. Vaccine. 2021;39(2):171-2.

66. Spatz DL. Advocate for the COVID-19 Vaccine for Pregnant and Breastfeeding Women. Mcn the American Journal of Maternal Child Nursing. 2021;46(3):178.

67. Rasmussen SA, Jamieson DJ. Pregnancy, Postpartum Care, and COVID-19 Vaccination in 2021. Jama. 2021;325(11):1099-100.

68. Qiu X, Bailey H, Thorne C. Barriers and Facilitators Associated With Vaccine Acceptance and Uptake Among Pregnant Women in High Income Countries: A Mini-Review. Front Immunol. 2021;12:626717.

69. Kalafat E, O'Brien P, Heath PT, Le Doare K, von Dadelszen P, Magee L, et al. Benefits and potential harms of COVID-19 vaccination during pregnancy: evidence summary for patient counseling. Ultrasound Obstet Gynecol. 2021;57(5):681-6.

70. Chahroudi A, Permar S. Will We Have the Tools to Address a Reemergent Zika Virus Epidemic? Ann Intern Med. 2021;174(5):708-9.

71. Cohen J. The line starts to form for a coronavirus vaccine. Science. 2020;369(6499):15-6.

72. Contreras MA, Arnegard ME, Chang MC, Hild S, Grieder F, Murphy SJ. Nonhuman primate models for SARS-CoV-2 Research: Managing demand for specific-pathogen-free (SPF) animals. Lab Anim (NY). 2021;50(8):200-1.

73. Christensen L, Damon S. Social Determinants of Health and Response to Disease Associated with Health Outcomes of American Indian and Alaska Native Patients. JAMA Netw Open. 2022;5(3):E224827.

74. Debiasi RL. Severe Acute Respiratory Syndrome-Coronavirus-2 Effects at the Maternal-Fetal Interface. J Infect Dis. 2022;225(5):745-7.

75. Ennab F, Babar MS, Khan AR, Mittal RJ, Nawaz FA, Essar MY, et al. Implications of social media misinformation on COVID-19 vaccine confidence among pregnant women in Africa. Clin Epidemiol Global Health. 2022;14.

76. Lin WS, Killeen D, Yang CY. Point-of-Care Ultrasound Is a Valuable Modality During Mass COVID-19 Vaccination Campaigns. J Ultrasound Med. 2022;41(5):1295-7.

77. Manca T, Baylis F, Munoz FM, Top KA. Prioritise research on vaccines for pregnant and breastfeeding women. Lancet. 2022;399(10328):890-3.

78. Wadman M. Studies reveal dangers of SARS-CoV-2 infection in pregnancy. Science. 2022;375(6578):253.

79. Walter K. Covid-19 and pregnancy. JAMA - Journal of the American Medical Association. 2022;327(8):790.

80. Mehaffey JH, Arnold M, Huffstetler E, Mehaffey RL, Quillian H, Mehaffey JH. Successful vertical transmission of SARS-CoV-2 antibodies after maternal vaccination. Birth. 2021;48(4):451-2.

81. Mangat C, Milosavljevic N. BNT162b2 Vaccination during Pregnancy Protects Both the Mother and Infant: Anti-SARS-CoV-2 S Antibodies Persistently Positive in an Infant at 6 Months of Age. Case Rep Pediatr. 2021;2021:6901131.

82. Soysal A, Bilazer C, Gönüllü E, Barın E, Çivilibal M. Cord blood antibody following maternal SARS-CoV-2 inactive vaccine (CoronaVac) administration during the pregnancy. Hum Vaccin Immunother. 2021;17(10):3484-6.

83. Gilbert P, Rudnick C. Newborn antibodies to SARS-CoV-2 detected in cord blood after maternal vaccination. MedRxiv. 2021.

84. Jaiswal V, Naz S, Ishak A, Batra N, Quinonez J, Mukherjee D, et al. A rare case of pediatric pancreatic pseudocyst. Clin Case Rep. 2022;10(5):e05879.

85. Uytenbogaardt A. COVID-19 vaccines and pregnancy. MA Healthcare London; 2021. p. 125-.

86. Yupatov EY, Maltseva L, Yusupova N, Safina L, Ignashina E, Kurmanbaev T, et al. To the question on vaccination of pregnant women during COVID-19 pandemic. Obstetrics, Gynecology and Reproduction. 2020;14(5):656-66.

87. Yan Z, Yang M, Lai CL. COVID-19 Vaccinations: A Comprehensive Review of Their Safety and Efficacy in Special Populations. Vaccines (Basel). 2021;9(10).

88. Sarwal Y, Sarwal T, Sarwal R. Prioritizing pregnant women for COVID-19 vaccination. Int J Gynaecol Obstet. 2021;155(1):57-63.

89. Quintana SM. We have Vaccine for COVID-19! What to Recommend for Pregnant Women? Rev Bras Ginecol Obstet. 2021;43(2):81-3.

90. Modi N, Ayres-de-Campos D, Bancalari E, Benders M, Briana D, Di Renzo GC, et al. Equity in coronavirus disease 2019 vaccine development and deployment. Am J Obstet Gynecol. 2021;224(5):423-7.

91. Jamieson DJ, Rasmussen SA. An update on COVID-19 and pregnancy. Am J Obstet Gynecol. 2022;226(2):177-86.

92. Hunter M, Moodley J, Moran N. Perspectives on COVID-19 vaccination for pregnant women in South Africa. Afr J Prim Health Care Fam Med. 2021;13(1):e1-e3.

93. Cinicola B, Conti MG, Terrin G, Sgrulletti M, Elfeky R, Carsetti R, et al. The Protective Role of Maternal Immunization in Early Life. Front Pediatr. 2021;9:638871.

94. Lapolla A, Dalfrà MG, Burlina S. Vaccination against COVID-19 infection: the need of evidence for diabetic and obese pregnant women. Acta Diabetol. 2021;58(12):1581-5.

95. Lv D, Peng J, Long R, Lin X, Wang R, Wu D, et al. Exploring the Immunopathogenesis of Pregnancy With COVID-19 at the Vaccination Era. Front Immunol. 2021;12:683440.

96. Moore KM, Suthar MS. Comprehensive analysis of COVID-19 during pregnancy. Biochem Biophys Res Commun. 2021;538:180-6.

97. Riviello C, Pontello V. Maternal and neonatal SARS-CoV-2 antibodies assessment after mRNA maternal vaccination in the third trimester of pregnancy. Int J Gynaecol Obstet. 2021;154(3):565-6.

98. Riley LE, Jamieson DJ. Inclusion of Pregnant and Lactating Persons in COVID-19 Vaccination Efforts. Ann Intern Med. 2021;174(5):701-2.

99. Rasmussen SA, Kelley CF, Horton JP, Jamieson DJ. Coronavirus Disease 2019 (COVID-19) Vaccines and Pregnancy: What Obstetricians Need to Know. Obstet Gynecol. 2021;137(3):408-14.

100. Pham A, Aronoff DM, Thompson JL. Maternal COVID-19, vaccination safety in pregnancy, and evidence of protective immunity. J Allergy Clin Immunol. 2021;148(3):728-31.

101. Munoz FM. Can We Protect Pregnant Women and Young Infants From COVID-19 Through Maternal Immunization? JAMA Pediatr. 2021;175(6):561-2.

102. Moro PL, Panagiotakopoulos L, Oduyebo T, Olson CK, Myers T. Monitoring the safety of COVID-19 vaccines in pregnancy in the US. Hum Vaccin Immunother. 2021;17(12):4705-13.

103. Martins I, Louwen F, Ayres-de-Campos D, Mahmood T. EBCOG position statement on COVID-19 vaccination for pregnant and breastfeeding women. Eur J Obstet Gynecol Reprod Biol. 2021;262:256-8.

104. Luxi N, Giovanazzi A, Capuano A, Crisafulli S, Cutroneo PM, Fantini MP, et al. COVID-19 Vaccination in Pregnancy, Paediatrics, Immunocompromised Patients, and Persons with History of Allergy or Prior SARS-CoV-2 Infection: Overview of Current Recommendations and Pre- and Post-Marketing Evidence for Vaccine Efficacy and Safety. Drug Saf. 2021;44(12):1247-69.

105. Girardelli S, Mullins E, Lees CC. COVID-19 and pregnancy: Lessons from 2020. Early Hum Dev. 2021;162:105460.

106. Girling J. COVID-19 vaccination in pregnancy. Drug Ther Bull. 2021;59(6):82.

107. Leik NKO, Ahmedy F, Guad RM, Baharuddin DMP. Covid-19 vaccine and its consequences in pregnancy: Brief review. Ann Med Surg (Lond). 2021;72:103103.

108. Joubert E, Kekeh AC, Amin CN. COVID-19 and novel mRNA vaccines in pregnancy: an updated literature review. Bjog. 2022;129(1):21-8.

109. Jorgensen SCJ, Burry L, Tabbara N. Role of maternal COVID-19 vaccination in providing immunological protection to the newborn. Pharmacotherapy. 2022;42(1):58-70.

110. Healy CM. COVID-19 in Pregnant Women and Their Newborn Infants. JAMA Pediatr. 2021;175(8):781-3.

111. Girardi G, Bremer AA. Scientific Evidence Supporting Coronavirus Disease 2019 (COVID-19) Vaccine Efficacy and Safety in People Planning to Conceive or Who Are Pregnant or Lactating. Obstet Gynecol. 2022;139(1):3-8.

112. Giles ML, Gunatilaka A, Palmer K, Sharma K, Roach V. Alignment of national COVID-19 vaccine recommendations for pregnant and lactating women. Bull World Health Organ. 2021;99(10):739-46.

113. Garg I, Shekhar R, Sheikh AB, Pal S. COVID-19 Vaccine in Pregnant and Lactating Women: A Review of Existing Evidence and Practice Guidelines. Infect Dis Rep. 2021;13(3):685-99.

114. Centor RM, Riley LE. Web Exclusive. Annals On Call - Pregnancy, Lactation, and the COVID-19 Vaccines. Ann Intern Med. 2021;174(3):Oc2.

115. Craig AM, Hughes BL, Swamy GK. Coronavirus disease 2019 vaccines in pregnancy. Am J Obstet Gynecol MFM. 2021;3(2):100295.

116. Etti M, Calvert A, Galiza E, Lim S, Khalil A, Le Doare K, et al. Maternal vaccination: a review of current evidence and recommendations. Am J Obstet Gynecol. 2022;226(4):459-74.

117. Duarte G, Coutinho CM, Rolnik DL, Quintana SM, Rabelo ESAC, Poon LC, et al. Perspectives on administration of COVID-19 vaccine to pregnant and lactating women: a challenge for low- and middle-income countries. AJOG Glob Rep. 2021;1(4):100020.

118. Donders GGG, Grinceviciene S, Haldre K, Lonnee-Hoffmann R, Donders F, Tsiakalos A, et al. ISIDOG Consensus Guidelines on COVID-19 Vaccination for Women before, during and after Pregnancy. J Clin Med. 2021;10(13).

119. Cypher RL. Maternal Vaccinations: Liability and Compensation. J Perinat Neonatal Nurs. 2021;35(2):116-9.

120. Chervenak FA, McCullough LB, Grünebaum A. Reversing physician hesitancy to recommend COVID-19 vaccination for pregnant patients. Am J Obstet Gynecol. 2021.

121. Chervenak FA, McCullough LB, Bornstein E, Johnson L, Katz A, McLeod-Sordjan R, et al. Professionally responsible coronavirus disease 2019 vaccination counseling of obstetrical and gynecologic patients. Am J Obstet Gynecol. 2021;224(5):470-8.

122. Schwartz DA, Graham AL. Potential Maternal and Infant Outcomes from (Wuhan) Coronavirus 2019-nCoV Infecting Pregnant Women: Lessons from SARS, MERS, and Other Human Coronavirus Infections. Viruses. 2020;12(2).

123. Sebghati M, Khalil A. Uptake of vaccination in pregnancy. Best Pract Res Clin Obstet Gynaecol. 2021;76:53-65.

124. Snook ML, Beigi RH, Legro RS, Paules CI. Should women undergoing in vitro fertilization treatment or who are in the first trimester of pregnancy be vaccinated immediately against COVID-19. Fertil Steril. 2021;116(1):16-24.

125. Volpe N, Luca Schera GB, Dall'Asta A, Di Pasquo E, Ghi T. COVID-19 in pregnancy: where are we now? J Perinat Med. 2021;49(6):637-42.

126. Principi N, Esposito S. Is the Immunization of Pregnant Women against COVID-19 Justified? Vaccines (Basel). 2021;9(9).

127. Pramanick A, Kanneganti A, Wong JLJ, Li SW, Dimri PS, Mahyuddin AP, et al. A reasoned approach towards administering COVID-19 vaccines to pregnant women. Prenat Diagn. 2021;41(8):1018-35.

128. Wang EW, Parchem JG, Atmar RL, Clark EH. SARS-CoV-2 Vaccination During Pregnancy: A Complex Decision. Open Forum Infect Dis. 2021;8(5):ofab180.

129. Stafford IA, Parchem JG, Sibai BM. The coronavirus disease 2019 vaccine in pregnancy: risks, benefits, and recommendations. Am J Obstet Gynecol. 2021;224(5):484-95.

130. Siddiqui S, Yassin HM. Effects of Covid-19 on pregnancy: An overview. Saudi Med J. 2021;42(7):798-800.

131. Şahin D, Tanaçan A, Webster SN, Moraloğlu Tekin Ö. Pregnancy and COVID-19: prevention, vaccination, therapy, and beyond. Turk J Med Sci. 2021;51(Si-1):3312-26.

132. Razzaghi H, Meghani M, Pingali C, Crane B, Naleway A, Weintraub E, et al. COVID-19 Vaccination Coverage Among Pregnant Women During Pregnancy - Eight Integrated Health Care Organizations, United States, December 14, 2020-May 8, 2021. MMWR Morb Mortal Wkly Rep. 2021;70(24):895-9.

133. Prochaska E, Jang M, Burd I. COVID-19 in pregnancy: Placental and neonatal involvement. Am J Reprod Immunol. 2020;84(5):e13306.

134. Deruelle P, Couffignal C, Sibiude J, Vivanti AJ, Anselem O, Luton D, et al. Prenatal care providers' perceptions of the SARS-Cov-2 vaccine for themselves and for pregnant women. PLoS One. 2021;16(9):e0256080.

135. Ghasemiyeh P, Mohammadi-Samani S, Firouzabadi N, Dehshahri A, Vazin A. A focused review on technologies, mechanisms, safety, and efficacy of available COVID-19 vaccines. Int Immunopharmacol. 2021;100:108162.

136. Mackin DW, Walker SP. The historical aspects of vaccination in pregnancy. Best Pract Res Clin Obstet Gynaecol. 2021;76:13-22.

137. Mahase E. Covid-19: Pregnant women should be offered Pfizer or Moderna vaccine, says UK advisory committee. Bmj. 2021;373:n1013.

138. Mirzaei R, Mohammadzadeh R, Mahdavi F, Badrzadeh F, Kazemi S, Ebrahimi M, et al. Overview of the current promising approaches for the development of an effective severe acute respiratory syndrome coronavirus 2 (SARS-CoV-2) vaccine. Int Immunopharmacol. 2020;88:106928.

139. Omar MAK, Jassat W, Brey Z, Parker S, Wadee M, Wadee S, et al. A call to action: Temporal trends of COVID-19 deaths in the South African Muslim community. S Afr Med J. 2021;111(9):13348.

140. Nowakowski F, Krajewska K, Klimek K, Wierzba W, Jakimiuk AJ. COVID-19 during pregnancy one year on - what lessons did we learn? Ginekol Pol. 2021;92(5):383-6.

141. Nana M, Nelson-Piercy C. COVID-19 in pregnancy. Clin Med (Lond). 2021;21(5):e446-e50.

142. Mohan S, Reagu S, Lindow S, Alabdulla M. COVID-19 vaccine hesitancy in perinatal women: a cross sectional survey. J Perinat Med. 2021;49(6):678-85.

143. Smagulova A, Uakhit R, Kiyan V. First Record of Alternaria alternata causing necrosis of Thuja (Thuja occidentalis) in Kazakhstan. Plant Dis. 2022.

144. Januszek SM, Faryniak-Zuzak A, Barnaś E, Łoziński T, Góra T, Siwiec N, et al. The Approach of Pregnant Women to Vaccination Based on a COVID-19 Systematic Review. Medicina (Kaunas). 2021;57(9).

145. Shook LL, Fallah PN, Silberman JN, Edlow AG. COVID-19 Vaccination in Pregnancy and Lactation: Current Research and Gaps in Understanding. Front Cell Infect Microbiol. 2021;11:735394.

146. Wong PKK, Lahiri M, Lye DC, Johnson D, Charles PGP. A vaccination update for rheumatologists-SARS-CoV-2, influenza and herpes zoster. Int J Rheum Dis. 2021;24(8):979-83.

147. Wilder-Smith A. COVID-19 in comparison with other emerging viral diseases: risk of geographic spread via travel. Trop Dis Travel Med Vaccines. 2021;7(1):3.

148. Wiersinga WJ, Rhodes A, Cheng AC, Peacock SJ, Prescott HC. Pathophysiology, Transmission, Diagnosis, and Treatment of Coronavirus Disease 2019 (COVID-19): A Review. Jama. 2020;324(8):782-93.

149. Behera BC, Mishra RR, Thatoi H. Recent biotechnological tools for diagnosis of corona virus disease: A review. Biotechnol Prog. 2021;37(1):e3078.

150. Chakraborty R, Parvez S. COVID-19: An overview of the current pharmacological interventions, vaccines, and clinical trials. Biochem Pharmacol. 2020;180:114184.

151. Dhillon P, Altmann D, Male V. COVID-19 vaccines: what do we know so far? Febs j. 2021;288(17):4996-5009.

152. Di Mascio D, Buca D, Berghella V, Khalil A, Rizzo G, Odibo A, et al. Counseling in maternal-fetal medicine: SARS-CoV-2 infection in pregnancy. Ultrasound Obstet Gynecol. 2021;57(5):687-97.

153. Kaur RJ, Dutta S, Bhardwaj P, Charan J, Dhingra S, Mitra P, et al. Adverse Events Reported From  COVID-19 Vaccine Trials: A Systematic Review. Indian J Clin Biochem. 2021;36(4):427-39.

154. Vress D. Future vaccines in pregnancy. Best Pract Res Clin Obstet Gynaecol. 2021;76:96-106.

155. Spini A, Giudice V, Brancaleone V, Morgese MG, De Francia S, Filippelli A, et al. Sex-tailored pharmacology and COVID-19: Next steps towards appropriateness and health equity. Pharmacol Res. 2021;173:105848.

156. Karrow NA, Shandilya UK, Pelech S, Wagter-Lesperance L, McLeod D, Bridle B, et al. Maternal COVID-19 Vaccination and Its Potential Impact on Fetal and Neonatal Development. Vaccines (Basel). 2021;9(11).

157. Celewicz A, Celewicz M, Michalczyk M, Woźniakowska-Gondek P, Krejczy K, Misiek M, et al. Pregnancy as a Risk Factor of Severe COVID-19. J Clin Med. 2021;10(22).

158. Yap C, Ali A, Prabhakar A, Prabhakar A, Pal A, Lim YY, et al. Comprehensive literature review on COVID-19 vaccines and role of SARS-CoV-2 variants in the pandemic. Ther Adv Vaccines Immunother. 2021;9:25151355211059791.

159. Dogra N, Ledesma-Feliciano C, Sen R. Developmental Aspects of SARS-CoV-2, Potential Role of Exosomes and Their Impact on the Human Transcriptome. J Dev Biol. 2021;9(4).

160. Arthurs AL, Jankovic-Karasoulos T, Roberts CT. COVID-19 in pregnancy: What we know from the first year of the pandemic. Biochim Biophys Acta Mol Basis Dis. 2021;1867(12):166248.

161. Lim RK, Kalagara S, Chen KK, Mylonakis E, Kroumpouzos G. Dermatology in a multidisciplinary approach with infectious disease and obstetric medicine against COVID-19. Int J Womens Dermatol. 2021;7(5):640-6.

162. Milota T, Strizova Z, Smetanova J, Sediva A. An immunologist's perspective on anti-COVID-19 vaccines. Curr Opin Allergy Clin Immunol. 2021;21(6):545-52.

163. Abu-Raya B, Madhi SA, Omer SB, Amirthalingam G, Giles ML, Flanagan KL, et al. Global Perspectives on Immunization Against SARS-CoV-2 During Pregnancy and Priorities for Future Research: An International Consensus Paper From the World Association of Infectious Diseases and Immunological Disorders. Front Immunol. 2021;12:808064.

164. Munnoli PM, Nabapure S, Yeshavanth G. Post-COVID-19 precautions based on lessons learned from past pandemics: a review. Z Gesundh Wiss. 2022;30(4):973-81.

165. Warrier S, Mohana Sundaram S, Varier L, Balasubramanian A. Stalling SARS-CoV2 infection with stem cells: can regenerating perinatal tissue mesenchymal stem cells offer a multi-tiered therapeutic approach to COVID-19? Placenta. 2022;117:161-8.

166. Carbone L, Di Girolamo R, Mappa I, Saccone G, Raffone A, Di Mascio D, et al. Worldwide beliefs among pregnant women on SARS-CoV-2 vaccine: a systematic review. Eur J Obstet Gynecol Reprod Biol. 2022;268:144-64.

167. Wiese MD, Berry MJ, Hissaria P, Darby JRT, Morrison JL. COVID-19: can we treat the mother without harming her baby? J Dev Orig Health Dis. 2022;13(1):9-19.

168. Riedel C, Rivera JC, Canedo-Marroquín G, Kalergis AM, Opazo MC. Respiratory viral infections during pregnancy: effects of SARS-CoV-2 and other related viruses over the offspring. J Dev Orig Health Dis. 2022;13(1):3-8.

169. Arora M, Lakshmi R. Vaccines - safety in pregnancy. Best Pract Res Clin Obstet Gynaecol. 2021;76:23-40.

170. Ursin RL, Klein SL. Sex Differences in Respiratory Viral Pathogenesis and Treatments. Annu Rev Virol. 2021;8(1):393-414.

171. COVID-19 vaccines. Drugs and Lactation Database (LactMed). Bethesda (MD): National Library of Medicine (US); 2006.

172. Mohseni Afshar Z, Babazadeh A, Janbakhsh A, Mansouri F, Sio TT, Sullman MJM, et al. Coronavirus disease 2019 (Covid-19) vaccination recommendations in special populations and patients with existing comorbidities. Rev Med Virol. 2022;32(3):e2309.

173. Abbas-Hanif A, Rezai H, Ahmed SF, Ahmed A. The impact of COVID-19 on pregnancy and therapeutic drug development. Br J Pharmacol. 2022;179(10):2108-20.

174. Liccardi G, Milanese M, Bilò MB, Martini M, Liccardi MV, Gargano D, et al. Lessons from peculiar cases of anaphylaxis: why allergists should be prepared for the unexpected. Eur Ann Allergy Clin Immunol. 2022;54(3):99-106.

175. Hsu AL, Johnson T, Phillips L, Nelson TB. Sources of Vaccine Hesitancy: Pregnancy, Infertility, Minority Concerns, and General Skepticism. Open Forum Infect Dis. 2022;9(3):ofab433.

176. Should pregnant women be vaccinated against COVID-19? Bull Acad Natl Med. 2021;205(5):439-40.

177. Abdoulaye MB, Oumarou B, Moussa H, Anya BPM, Didier T, Nsiari-Muzeyi BJ, et al. The impact of the COVID-19 pandemic on health service utilisation in the city of Niamey: A study conducted in 17 health care facilities from january to june 2020. Pan Afr Med J. 2021;39.

178. Adamyan LV, Filippov OS, Kharchenko EI, Stepanian AA, Aznaurova YB, Konysheva OV. Vaccination against COVID-19 in pregnancy is safe and effective (Literature review). Rus J Hum Reprod. 2021;27(5).

179. Adamyan LV, Filippov OS, Stepanian AA, Aznaurova Y, Konysheva OV. Covid-19 vaccines and pregnancy (Literature review). Rus J Hum Reprod. 2021;27(3-2):5-14.

180. Alejandra Taborda R, Murillo DA, Carolina Moreno L, Paula Andrea Taborda R, Fuquen M, Díaz PA, et al. Analysis of budgetary impact of COVID-19 vaccination in Latin America. Rev Panam Salud Publica Pan Am J Public Health. 2022;46.

181. Álvarez García FJ, Cilleruelo Ortega MJ, Álvarez Aldeán J, Garcés-Sánchez M, Garrote Llanos E, Iofrío de Arce A, et al. Immunisation schedule of the Pediatric Spanish Association: 2022 recommendations. An Pediatr. 2022;96(1):59.e1-.e10.

182. Ayesa-Arriola R, López-Díaz Á, Ruiz-Veguilla M, Leza JC, Saura LF, Crespo-Facorro B. COVID-19 as a unique opportunity to unravel the link between prenatal maternal infection, brain development and neuropsychiatric disorders in offspring. Rev Psiquiatr Salud Ment. 2021;14(1):1-3.

183. Blanco MG. COVID-19, vaccines and pregnancy. Rev Obstet Ginecol Venez. 2020;80(4):263-7.

184. de Carvajal ACC. Considerations about COVID-19 vaccines in pregnant and nursing mothers. Gac Med Caracas. 2021;129(2):454-63.

185. Dolgushina NV, Drapkina YS, Krechetova LV, Ivanets TY, Menzhinskaya IV, Gus AI, et al. Gam-covid-vac (Sputnik v) vaccine has no adverse effect on ovarian reserve in reproductive-age women. Akush Ginekol. 2021;2021(7):81-6.

186. Lepigeon K, Eberhardt CS, Favre G, Baud D, De Tejada BM. Pregnancy and COVID-19: drugs and vaccine guidelines in 2021. Rev Med Suisse. 2022;18(767):165-8.

187. Li P, Liu H, Zou Y, Fang M, Chen Y, Zhang W. How to think about severe acute respiratory syndrome coronavirus 2 vaccination for pregnant and lactating women in China. J Cent South Univ Med Sci. 2021;46(12):1386-91.

188. Miauton A, Besson J, Muller Y, Genton B. mRNA Covid vaccines: What do we know eight months after deployment. Rev Med Suisse. 2021;17(758):1910-4.

189. Olcina MJE, Astorga GF, Toledo BJ, Páez EO, Cáceres JRC. Efficacy and safety of SARS-CoV-2 vaccines. Pediatria Aten Primaria. 2022;24(93):e171-e82.

190. Pan HX, Huang BY, Deng Y, Chu K, Hu JL, Zhu DD, et al. Immunogenicity and safety of a booster vaccination with an inactivated severe acute respiratory syndrome coronavirus 2 vaccine in adults aged 18 to 59 years. Zhonghua Yi Xue Za Zhi. 2022;102(4):279-85.

191. Qiao J, Expert Group for Beijing Human Assisted Reproductive Technology Center For Quality C, Improvement. COVID-19 vaccination strategy for planning pregnancy and assisted reproductive technology treatment: expert recommendations. Chinese J Reprod Contracep. 2021;41(4):296-9.

192. Vinelli-Arzubiaga D, Marquez-Bravo AW, Ortega IGA, Rodriguez-Alarcón JF, Arias-Chavez D, Vilela-Estrada MA, et al. Acceptance of COVID-19 vaccination among pregnant Peruvian women: Attitudes and associated factors. Bol Malariol Salud Ambient. 2021;61:45-52.

193. Yupatov EY, Maltseva LI, Yusupova NZ, Safina LZ, Ignashina EG, Kurmanbaev TE, et al. To the question on vaccination of pregnant women during COVID-19 pandemic. Obstetrics, Gynecology and Reproduction. 2020;14(5):656-67.

194. Zhibin P, Luzhao F, Dayan W. Technical guidelines for seasonal influenza vaccination in China (2021-2022). Chin J Endemiol. 2021;42(10):1722-49.

195. Hayakawa S, Komine-Aizawa S, Takada K, Kimura T, Yamada H. Anti-SARS-CoV-2 vaccination strategy for pregnant women in Japan. J Obstet Gynaecol Res. 2021;47(6):1958-64.

196. Sculli MA, Formoso G, Sciacca L. COVID-19 vaccination in pregnant and lactating diabetic women. Nutr Metab Cardiovasc Dis. 2021;31(7):2151-5.

197. Saso A, Skirrow H, Kampmann B. Impact of COVID-19 on Immunization Services for Maternal and Infant Vaccines: Results of a Survey Conducted by Imprint-The Immunising Pregnant Women and Infants Network. Vaccines (Basel). 2020;8(3).

198. Seven days in medicine: 26 May to 2 June 2021. The BMJ. 2021;373.

199. Cohen J. 'Landmark' African vaccine trial faces impasse. Science. 2021;372(6547):1135-6.

200. Huckriede ALW. COVID-19 update: COVID-19 vaccination and pregnancy. Ned Tijdschr Geneeskd. 2021;165(20).

201. Lurie N, Experton B. How to Leverage the Medicare Program for a COVID-19 Vaccination Campaign. JAMA - Journal of the American Medical Association. 2021;325(1):21-2.

202. Newman C, Henderson C, Laraque-Arena D. COVID-19 and Pregnancy: A public health, evidence-based approach. J Natl Med Assoc. 2022;114(1):42-6.

203. Rubin R. Trying to Block SARS-CoV-2 Transmission with Intranasal Vaccines. JAMA - Journal of the American Medical Association. 2021;326(17):1661-3.

204. Rubin R. Pregnant People's Paradox-Excluded from Vaccine Trials despite Having a Higher Risk of COVID-19 Complications. JAMA - Journal of the American Medical Association. 2021;325(11):1127-8.

205. Shah A, Challener DW, O'Horo JC, Badley AD. Vaccination Safety: Don't Toss the Champagne With the Cork. Mayo Clin Proc. 2021;96(7):1712-3.

206. Rubin EJ, Baden LR, Walensky RP, Morrissey S. Audio Interview: Covid-19 Vaccines and Pregnancy - A Conversation with CDC Director Rochelle Walensky. N Engl J Med. 2021;384(16):e73.

207. Male V. Author Correction: Are COVID-19 vaccines safe in pregnancy? Nat Rev Immunol. 2021;21(4):268.

208. Erratum: mRNA COVID-19 Vaccines in Pregnant Women (New England Journal of Medicine (2021) 384 (2342-2343) DOI: 10.1056/NEJMe2107070). New Engl J Med. 2021;385(16):1536.

209. Erratum: Preliminary Findings of mRNA COVID-19 Vaccine Safety in Pregnant Persons (N Engl J Med (2021) 384 (2273-2282) DOI: 10.1056/NEJMoa2104983). New Engl J Med. 2021;385(16):1536.

210. Erratum: Coronavirus Disease 2019 (COVID-19) Vaccines and Pregnancy What Obstetricians Need to Know: Correction (Obstetrics and gynecology (2021) 137 3 (408-414)). Obstetrics and gynecology. 2021;137(5):962.

211. Errata: “Interim Estimates of COVID-19 Vaccine Effectiveness Against COVID-19–Associated Emergency Department or Urgent Care Clinic Encounters and Hospitalizations Among Adults During SARS-CoV-2 B.1.617.2 (Delta) Variant Predominance — Nine States, June–August 2021,” (Morbidity and Mortality Weekly Report, 70(37), (1291–1293), (10.15585/mmwr.mm7037e2)). MMWR Recomm Rep. 2021;70(49):1717-.

212. Erratum: Interim Estimates of COVID-19 Vaccine Effectiveness against COVID-19–Associated Emergency Department or Urgent Care Clinic Encounters and Hospitalizations among Adults during SARS-CoV-2 B.1.617.2 (Delta) Variant Predominance — Nine States, June–August 2021 (Morbidity and Mortality Weekly Report 70:37 (1293) DOI: 10.1101/2021.08.06.21261707v2). Morb Mortal Wkly Rep. 2021;70(49):1717.

213. Male V. Author Correction: Are COVID-19 vaccines safe in pregnancy? (Nature Reviews Immunology, (2021), 21, 4, (200-201), 10.1038/s41577-021-00525-y). Nat Rev Immunol. 2021;21(4):268.

214. Pregnant women fare well with covid vaccine, survey finds. US Pharm. 2021;46(9):4.

215. NASPAG Position Statement on COVID-19 Vaccines and Gynecologic Concerns in Adolescents and Young Adults. J Pediatr Adolesc Gynecol. 2021;34(4):439-40.

216. Abbas-Hanif A, Modi N, Smith SK, Majeed A. Covid-19 treatments and vaccines must be evaluated in pregnancy. The BMJ. 2021;375.

217. Abbasi K. Folic acid supplementation and the complexities of blame. The BMJ. 2022;376.

218. Adnani QES, O'Connell MA, Homer CSE. Advocating for midwives in low-to-middle income countries in the COVID-19 pandemic. Women Birth. 2021;34(6):501-2.

219. Ahlberg M. Editorial. Sex Reprod Healthc. 2022;31.

220. Allahbadia G. Will Procreation Ever Be The Same After COVID-19? J Obstet Gynecol India. 2021;71.

221. Clain E, Johnson J, Roeca C. Triggered with COVID: What are my chances, Doc? Fertil Steril. 2022;117(4):781-2.

222. Dangal G. COVID-19 Vaccination: Urgent Efforts Must Now Be Made to Ensure that Pregnant Women Are Fast Tracked and Prioritized. Kathmandu Univ Med J. 2021;19(75):285-6.

223. Delara M, Sadarangani M. Immunization in pregnancy to protect pregnant people and their newborns against COVID-19. Expert Rev Vaccines. 2022;21(5):593-5.

224. Dermody TS, Dimaio D, Enquist LW. Vaccine Safety, Efficacy, and Trust Take Time. Annu Rev Virol. 2021;8:III-V.

225. Dol J, Dennis CL. Striving for evidence-based health care with eHealth and technology in a time of half-truths and disinformation. JBI Evid Synth. 2021;19(10):2474-5.

226. Elston DM. This month in JAAD: January 2022. J Am Acad Dermatol. 2022;86(1):39.

227. Kharbanda EO, Vazquez-Benitez G. COVID-19 mRNA Vaccines during Pregnancy: New Evidence to Help Address Vaccine Hesitancy. JAMA - Journal of the American Medical Association. 2022;327(15):1451-3.

228. Legro RS. Coronavirus disease 2019 vaccination in women seeking pregnancy or in early pregnancy: What is there to debate? Fertil Steril. 2021;116(1):15.

229. Lim BHSSS. Immunisation in pregnancy. Best Pract Res Clin Obstet Gynaecol. 2021;76:1-2.

230. Male V. Menstrual changes after covid-19 vaccination. The BMJ. 2021;374.

231. Nadimpally S. Pregnancy and Covid vaccine trials: Gender justice compromised. Indian J Med Ethics. 2021;VI(3):1-8.

232. Pham A, Aronoff DM, Thompson JL. Maternal COVID-19, vaccination safety in pregnancy, and evidence of protective immunity. J Allergy Clin Immunol. 2021;148(3):728-31.

233. Rahman SU. NEONATAL PERINATAL COVID-19. J Postgrad Med Inst. 2021;35(2):61-2.

234. Riley LE. MRNA Covid-19 vaccines in pregnant women. New Engl J Med. 2021;384(24):2342-3.

235. Rubin EJ, Baden LR, Morrissey S. Audio interview: A new look at covid-19 vaccine boosters. New Engl J Med. 2021;385(17):E72.

236. Snook ML, Beigi RH, Legro RS, Paules CI. Should women undergoing in vitro fertilization treatment or who are in the first trimester of pregnancy be vaccinated immediately against COVID-19. Fertil Steril. 2021;116(1):16-24.

237. The Lancet C, Adolescent H. A roadmap of recovery for the COVID generation. Lancet Child Adolesc Health. 2022;6(4):215.

238. The Lancet Digital H. Pregnancy in a pandemic: inequalities in maternal health. Lancet Digit Heal. 2022;4(2):e75.

239. Awasthi S, Knox JJ, Desmond A, Alameh MG, Gaudette BT, Lubinski JM, et al. Trivalent nucleoside-modified mRNA vaccine yields durable memory B cell protection against genital herpes in preclinical models. J Clin Invest. 2021;131(23).

240. Fikadu Y, Yeshaneh A, Melis T, Mesele M, Anmut W, Argaw M. COVID-19 Preventive Measure Practices and Knowledge of Pregnant Women in Guraghe Zone Hospitals. Int J Womens Health. 2021;13:39-50.

241. Bowman CJ, Bouressam M, Campion SN, Cappon GD, Catlin NR, Cutler MW, et al. Lack of effects on female fertility and prenatal and postnatal offspring development in rats with BNT162b2, a mRNA-based COVID-19 vaccine. Reprod Toxicol. 2021;103:28-35.

242. Zhang G, Li B, Yoo D, Qin T, Zhang X, Jia Y, et al. Animal coronaviruses and SARS-CoV-2. Transbound Emerg Dis. 2021;68(3):1097-110.

243. Bleicher I, Kadour-Peero E, Sagi-Dain L, Sagi S. Early exploration of COVID-19 vaccination safety and effectiveness during pregnancy: interim descriptive data from a prospective observational study. Vaccine. 2021;39(44):6535-8.

244. Ciapponi A, Bardach A, Mazzoni A, Alconada T, Anderson SA, Argento FJ, et al. Safety of components and platforms of COVID-19 vaccines considered for use in pregnancy: A rapid review. Vaccine. 2021;39(40):5891-908.

245. Giersing BK, Vekemans J, Nava S, Kaslow DC, Moorthy V, the WHOPDfVAC. Report from the World Health Organization's third Product Development for Vaccines Advisory Committee (PDVAC) meeting, Geneva, 8–10th June 2016. Vaccine. 2019;37(50):7315-27.

246. Gurwith M, Condit RC, Excler JL, Robertson JS, Kim D, Fast PE, et al. Brighton Collaboration Viral Vector Vaccines Safety Working Group (V3SWG) standardized template for collection of key information for benefit-risk assessment of live-attenuated viral vaccines. Vaccine. 2020;38(49):7702-7.

247. Maykin MM, Heuser C, Feltovich H, with the Society for Maternal-Fetal Medicine Health Policy Advocacy C. Pregnant people deserve the protection offered by SARS-CoV-2 vaccines. Vaccine. 2021;39(2):171-2.

248. Moro PL, Olson CK, Clark E, Marquez P, Strid P, Ellington S, et al. Post-authorization surveillance of adverse events following COVID-19 vaccines in pregnant persons in the vaccine adverse event reporting system (VAERS), December 2020 – October 2021. Vaccine. 2022;40(24):3389-94.

249. Saleh OA, Halperin O. Influenza virus vaccine compliance among pregnant women during the COVID-19 pandemic (pre-vaccine era) in Israel and future intention to uptake BNT162b2 mRNA COVID-19 vaccine. Vaccine. 2022;40(13):2099-106.

250. Simmons LA, Whipps MDM, Phipps JE, Satish NS, Swamy GK. Understanding COVID-19 vaccine uptake during pregnancy: ‘Hesitance’, knowledge, and evidence-based decision-making. Vaccine. 2022;40(19):2755-60.

251. Wainstock T, Yoles I, Sergienko R, Sheiner E. Prenatal maternal COVID-19 vaccination and pregnancy outcomes. Vaccine. 2021;39(41):6037-40.

252. Zimmerman RK. Helping patients with ethical concerns about COVID-19 vaccines in light of fetal cell lines used in some COVID-19 vaccines. Vaccine. 2021;39(31):4242-4.

253. Brandt JS, Fell DB. SARS-CoV-2 infection in pregnancy: Lessons learned from the first pandemic wave. Paediatr Perinat Epidemiol. 2021;35(1):34-6.

254. Brown LK, Freemantle N, Breuer J, Dehbi HM, Chowdhury K, Jones G, et al. Early antiviral treatment in outpatients with COVID-19 (FLARE): a structured summary of a study protocol for a randomised controlled trial. Trials. 2021;22(1):193.

255. Ceulemans M, Foulon V, Panchaud A, Winterfeld U, Pomar L, Lambelet V, et al. Vaccine Willingness and Impact of the COVID-19 Pandemic on Women's Perinatal Experiences and Practices-A Multinational, Cross-Sectional Study Covering the First Wave of the Pandemic. Int J Environ Res Public Health. 2021;18(7).

256. Chakraborty S, Gonzalez JC, Sievers BL, Mallajosyula V, Chakraborty S, Dubey M, et al. Structurally and functionally distinct early antibody responses predict COVID-19 disease trajectory and mRNA vaccine response. bioRxiv. 2021.

257. Cool K, Gaudreault NN, Morozov I, Trujillo JD, Meekins DA, McDowell C, et al. Infection and transmission of ancestral SARS-CoV-2 and its alpha variant in pregnant white-tailed deer. bioRxiv. 2021.

258. Corchado-Garcia J, Zemmour D, Hughes T, Bandi H, Cristea-Platon T, Lenehan P, et al. Analysis of the Effectiveness of the Ad26.COV2.S Adenoviral Vector Vaccine for Preventing COVID-19. JAMA Netw Open. 2021;4(11):e2132540.

259. Corrao G, Cantarutti A, Monzio Compagnoni M, Franchi M, Rea F. Change in healthcare during Covid-19 pandemic was assessed through observational designs. J Clin Epidemiol. 2022;142:45-53.

260. Godeau D, Petit A, Richard I, Roquelaure Y, Descatha A. Return-to-work, disabilities and occupational health in the age of COVID-19. Scand J Work Environ Health. 2021;47(5):408-9.

261. Murphy WJ. The urgent need for more basic research on SARS-Cov2 infection and vaccines in assessing potential psychoneurological effects using maternal immune activation (MIA) and other preclinical modeling. Brain Behav Immun. 2021;97:1-3.

262. Chandi A, Jain N. State of assisted reproduction technology in the coronavirus disease 2019 era and consequences on human reproductive system. Biol Reprod. 2021;105(4):808-21.

263. Emanoil AR, Stochino Loi E, Feki A, Ben Ali N. Focusing Treatment on Pregnant Women With COVID Disease. Front Glob Womens Health. 2021;2:590945.
