# Supplementary material for: Maternal and neonatal safety outcomes after SAR-CoV-2 vaccination during pregnancy: a systematic review and meta-analysis
Source: BMC Pregnancy Childbirth. 2022 Jul 21;22:581. doi: 10.1186/s12884-022-04884-9 (PMC9302221; doi:10.1186/s12884-022-04884-9)
Supplement: Supplementary file 1 — Additional file 1: Supplemental Figure 1. The effect of Mido(L)-ATRA on the content of Annexin V+ cells. HL-60 cells were treated with 0.25 μM modistaurin (M(L)) and/or 0.1 μM ATRA for 6 d. HL-60Res and U937 cells were treated with 0.1 μM modistaurin (M(L)) and/or 1 μM ATRA for 12 and 8 d, respectively. (A) The column graph of the content of Annexin V+ cells in three cell lines. Each value represents the mean ± SD of three independent measurements. (B) Representative scattered plotgrams of Annexin V expression. Results were representative among three independent experiments. Supplemental Figure 2. The effect of Mido(H)-ATRA on the content of CD11b+ cells. Cells were treated with 0.5 μM midostaurin (M(H)) and/or ATRA for 2 d. (A) The column graph of CD11b expression in three cell lines. Each value represents the mean ± SD of three independent measurements. ***P<0.005, versus DMSO-treated cells. (B) Representative histograms of CD11b expression with high dose midostaurin and/or ATRA. Results were representative among three independent experiments. Supplemental Figure 3. Most membranes were cut prior to hybridization. Original blots of the immunoblot detection shown in Fig 2A-Fig 2B, Fig 3D, Fig 4A-Fig 4C, Fig 5A and Fig 5E. [file 12884_2022_4884_MOESM1_ESM.zip › Supplementary File 3.docx]

Supplementary File 3A: Quality assessment for cohort study based on Newcastle-Ottawa-Scale (NOS)

| Study ID | Selection | | | | Comparability | Outcome | | | Quality score |
| --- | --- | --- | --- | --- | --- | --- | --- | --- | --- |
|  | Representativeness of the exposed cohort | Selection of the non-exposed cohort | Ascertainment of exposed | Demonstration that outcome of interest was not present at the start of the study | Comparability of cohorts on the basis of the design or analysis | Assessment of outcome | Was follow up long enough for outcomes to occur | Adequacy of follow up of cohorts |  |
| Beharier 2021 | * | * | * | * | ** | * | * |  | Good quality |
| Blakeway 2021 |  | * | * | * | * | * | * | * | Good quality |
| Collier 2021 |  | * | * | * | * | * | * | * | Good quality |
| Dagan 2021 |  | * | * | * |  | * | * |  | Poor quality |
| Kharbanda 2021 | * | * | * | * | ** | * | * | * | Good quality |
| Rottenstreich 2021 | * | * | * | * | * | * | * |  | Good quality |
| Shanes 2021 |  | * | * | * | * | * | * | * | Good quality |
| Theiler 2021 |  | * | * | * | * | * | * | * | Good quality |
| Wainstock 2021 | * | * | * | * | * | * | * |  | Good quality |

Supplementary File 3B: Quality assessment template for case-control study based on Newcastle-Ottawa-Scale (NOS)

| Study ID | Selection | | | | Comparability | Outcome Or Exposure | | | Quality score |
| --- | --- | --- | --- | --- | --- | --- | --- | --- | --- |
|  | Is the case definition adequate | Representativeness of the cases | Selection of Controls | Definition of Controls | Comparability of cases and controls on the basis of the design or analysis | Ascertainment of exposure | Same method of ascertainment for cases and controls | Non-Response rate |  |
| Butt 2021 | * | * |  | * | * | * | * |  | Good quality |
|  |  |  |  |  |  |  |  |  |  |

Supplementary File 3C: the authors' opinions and explanations regarding ROB of included RCTs.

| Study ID | Random sequence generation (selection bias) | Allocation concealment (selection bias) | Blinding of participants and personnel (performance bias) | Blinding of outcome assessment (Detection bias) | Incomplete outcome data (attrition bias) | Selective reporting (reporting bias) | Other Bias |  |
| --- | --- | --- | --- | --- | --- | --- | --- | --- |
| Pfizer BioNTech C4591001 | Low | Low | Low | Low | Low | Low | High |  |
|  |  |  |  |  |  |  |  |  |
| Moderna mRNA-1273-P301 | Unclear | Unclear | Low | Unclear | Low | Low | High |  |
| COV003 (Brazil) | Unclear | Unclear | Unclear | Unclear | Unclear | Low | High |  |
