# Supplementary material for: Maternal and neonatal safety outcomes after SAR-CoV-2 vaccination during pregnancy: a systematic review and meta-analysis
Source: BMC Pregnancy Childbirth. 2022 Jul 21;22:581. doi: 10.1186/s12884-022-04884-9 (PMC9302221; doi:10.1186/s12884-022-04884-9)
Supplement: Supplementary file 1 — Additional file 1: Supplemental Figure 1. The effect of Mido(L)-ATRA on the content of Annexin V+ cells. HL-60 cells were treated with 0.25 μM modistaurin (M(L)) and/or 0.1 μM ATRA for 6 d. HL-60Res and U937 cells were treated with 0.1 μM modistaurin (M(L)) and/or 1 μM ATRA for 12 and 8 d, respectively. (A) The column graph of the content of Annexin V+ cells in three cell lines. Each value represents the mean ± SD of three independent measurements. (B) Representative scattered plotgrams of Annexin V expression. Results were representative among three independent experiments. Supplemental Figure 2. The effect of Mido(H)-ATRA on the content of CD11b+ cells. Cells were treated with 0.5 μM midostaurin (M(H)) and/or ATRA for 2 d. (A) The column graph of CD11b expression in three cell lines. Each value represents the mean ± SD of three independent measurements. ***P<0.005, versus DMSO-treated cells. (B) Representative histograms of CD11b expression with high dose midostaurin and/or ATRA. Results were representative among three independent experiments. Supplemental Figure 3. Most membranes were cut prior to hybridization. Original blots of the immunoblot detection shown in Fig 2A-Fig 2B, Fig 3D, Fig 4A-Fig 4C, Fig 5A and Fig 5E. [file 12884_2022_4884_MOESM1_ESM.zip › Supplementary File 4.docx]

**The quality of evidence of the analyzed outcomes using the GRADE tool.**

**Author(s):** Abdulrahman Ibrahim Hagrass

**Question:** Should SAR-CoV-2 vaccine be used in women during pregnancy?

**Setting:**

**Bibliography:**

| **Certainty assessment** | | | | | | | | | | | **№ of patients** | | **Effect** | | | **Certainty** | | **Importance** | |  |
| --- | --- | --- | --- | --- | --- | --- | --- | --- | --- | --- | --- | --- | --- | --- | --- | --- | --- | --- | --- | --- |
| **№ of studies** | | **Study design** | | **Risk of bias** | | **Inconsistency** | | **Indirectness** | **Imprecision** | **Other considerations** | **[SAR-CoV-2 vaccine]** | **Control** | **Relative (95% CI)** | **Absolute (95% CI)** | |  |  |  |  |  |
| **Miscarriage** | | | | | | | | | | | | | | | | | | | |  |
| 5 | observational studies | | serious^a^ | | not serious | | not serious | | serious | none | 14/898 (1.6%) | 9/2955 (0.3%) | **RR 1.23** (0.54 to 2.78) | | **1 more per 1,000** (from 1 fewer to 5 more) | | ⨁◯◯◯ Very low | | CRITICAL | |
| **Neonatal unit admission** | | | | | | | | | | | | | | | | | | | | |
| 4 | observational studies | | not serious | | not serious | | not serious | | serious | none | 41/1077 (3.8%) | 81/3390 (2.4%) | **RR 0.98** (0.67 to 1.43) | | **0 fewer per 1,000** (from 8 fewer to 10 more) | | ⨁◯◯◯ Very low | | CRITICAL | |
| **Apgar ≤ 7 at 5 min** | | | | | | | | | | | | | | | | | | | | |
| 3 | observational studies | | not serious | | not serious | | not serious | | serious | none | 26/1765 (1.5%) | 95/6411 (1.5%) | **RR 0.86** (0.54 to 1.37) | | **2 fewer per 1,000** (from 7 fewer to 5 more) | | ⨁◯◯◯ Very low | | CRITICAL | |
| **Composite adverse neonatal outcome** | | | | | | | | | | | | | | | | | | | | |
| 2 | observational studies | | not serious | | not serious | | not serious | | not serious | none | 63/852 (7.4%) | 212/2925 (7.2%) | **RR 0.73** (0.55 to 0.96) | | **20 fewer per 1,000** (from 33 fewer to 3 fewer) | | ⨁⨁◯◯ Low | | CRITICAL | |
| **Postpartum hemorrhage** | | | | | | | | | | | | | | | | | | | | |
| 3 | observational studies | | not serious | | not serious | | not serious | | serious | none | 75/1758 (4.3%) | 174/4948 (3.5%) | **RR 0.84** (0.65 to 1.09) | | **6 fewer per 1,000** (from 12 fewer to 3 more) | | ⨁◯◯◯ Very low | | CRITICAL | |
| **Cesarean** | | | | | | | | | | | | | | | | | | | | |
| 4 | observational studies | | not serious | | not serious | | not serious | | not serious | none | 378/1898 (19.9%) | 1393/6810 (20.5%) | **RR 1.18** (1.06 to 1.31) | | **37 more per 1,000** (from 12 more to 63 more) | | ⨁⨁◯◯ Low | | IMPORTANT | |
| **Instrumental OR Vacuum-assisted delivery** | | | | | | | | | | | | | | | | | | | | |
| 4 | observational studies | | not serious | | serious^b^ | | not serious | | serious | none | 79/1898 (4.2%) | 309/6810 (4.5%) | **RR 0.94** (0.57 to 1.56) | | **3 fewer per 1,000** (from 20 fewer to 25 more) | | ⨁◯◯◯ Very low | | IMPORTANT | |

**CI:** confidence interval; **MD:** mean difference; **RR:** risk ratio

#### Explanations

a. Potential limitations are likely to lower confidence in the estimate of effect.

b. Unexplained heterogeneity
